# Elucidation of the Gemcitabine Transporters of *Escherichia coli* K-12 and Gamma-Proteobacteria Linked to Gemcitabine-Related Chemoresistance

**DOI:** 10.3390/ijms25137012

**Published:** 2024-06-27

**Authors:** Nikoleta Iosifidou, Eleni Anagnostopoulou, Maria Botou, Eirini Kalfa, Ekaterini Tatsaki, Stathis Frillingos

**Affiliations:** 1 Laboratory of Biological Chemistry, Department of Medicine, School of Health Sciences, University of Ioannina, 45110 Ioannina, Greece; nikoletaiosifidou12@gmail.com (N.I.); anagnostopoulou.eleni@outlook.com (E.A.); mpotoumaria@yahoo.gr (M.B.); eirinikalfa347@gmail.com (E.K.); ctatsaki@yahoo.gr (E.T.); 2 University Research Center of Ioannina (URCI), Institute of Biosciences, 45110 Ioannina, Greece

**Keywords:** gemcitabine, chemoresistance, gamma-proteobacteria, *Escherichia coli* K-12, nucleoside transporters

## Abstract

Gemcitabine (2′,2′-difluoro-2′-deoxycytidine), a widely used anticancer drug, is considered a gold standard in treating aggressive pancreatic cancers. Gamma-proteobacteria that colonize the pancreatic tumors contribute to chemoresistance against gemcitabine by metabolizing the drug to a less active and deaminated form. The gemcitabine transporters of these bacteria are unknown to date. Furthermore, there is no complete knowledge of the gemcitabine transporters in *Escherichia coli* or any other related proteobacteria. In this study, we investigate the complement of gemcitabine transporters in *E. coli* K-12 and two common chemoresistance-related bacteria (*Klebsiella pneumoniae* and *Citrobacter freundii*). We found that *E. coli* K-12 has two high-affinity gemcitabine transporters with distinct specificity properties, namely, NupC and NupG, whereas the gemcitabine transporters of *C. freundii* and *K. pneumoniae* include the NupC and NupG orthologs, functionally indistinguishable from their counterparts, and, in *K. pneumoniae*, one additional NupC variant, designated KpNupC2. All these bacterial transporters have a higher affinity for gemcitabine than their human counterparts. The highest affinity (*K*_M_ 2.5–3.0 μΜ) is exhibited by NupGs of the bacteria-specific nucleoside-H^+^ symporter (NHS) family followed by NupCs (*K*_M_ 10–13 μΜ) of the concentrative nucleoside transporter (CNT) family, 15–100 times higher than the affinities reported for the human gemcitabine transporter hENT1/SLC29A1, which is primarily associated with gemcitabine uptake in the pancreatic adenocarcinoma cells. Our results offer a basis for further insight into the role of specific bacteria in drug availability within tumors and for understanding the structure–function differences of bacterial and human drug transporters.

## 1. Introduction

Purine and pyrimidine nucleobases/nucleosides are essential to life as we know it given that they are the coding building blocks of genetic information and core moieties of molecules with fundamental roles in information flow, signaling, and metabolism [1,2,3].

Based on their role as antimetabolites, nucleobase/nucleoside analogs have long been used as antimicrobial, antiviral, or anticancer drugs [4]. Such analogs hijack the nucleotide metabolism and inhibit key nucleotide-salvage/interconversion enzymes or become incorporated into DNA or RNA, leading to cytotoxicity.

Despite the plenitude of knowledge on intracellular antimetabolite metabolism, the cellular uptake of nucleobase/nucleoside antimetabolites at the level of the membrane is still understudied, especially in bacteria. In particular, regarding the cellular uptake of anticancer antimetabolites, which is important for understanding variations in drug effectiveness and chemoresistance, research has focused almost entirely on the human genome-encoded transporters of tumor cells [5,6] and rarely, if at all, on bacterial drug-transporting counterparts in the associated tumor microenvironment.

Bacteria in the tumor microenvironment can greatly affect the availability and toxicity of antimetabolites to the cancer cells and contribute to chemoresistance. A prominent example concerns gemcitabine (2′,2′-difluoro-2′-deoxycytidine) (dFdC), a cytidine analog commonly used in cancer therapy and especially as a frontline drug for pancreatic cancer [7]. The bacteria-mediated metabolism of gemcitabine has been implicated in enhanced chemoresistance in pancreatic ductal adenocarcinoma [8]. The phenomenon is also linked to the active transport of the drug by the bacteria based on indirect evidence from the preincubation of gemcitabine with *Escherichia coli* K-12 devoid of the nucleoside-related transporter gene *nupC* and the use of the cell-free supernatant in a pancreatic adenocarcinoma cell line culture [8]. However, the transporters responsible for the uptake of gemcitabine by the tumor-associated bacteria have not been elucidated to date.

Bacteria colonizing pancreatic tumors are diverse, but gamma-proteobacteria of certain genera are common in these populations [8,9] and possess a so-called long isoform of cytidine deaminase (CDD_L_), which has been found to be correlated with the phenomenon of bacteria-mediated chemoresistance [8]. The gemcitabine transporters in these bacteria are unknown but, based on phylogenetic considerations, they might belong to the concentrative nucleoside transporter (CNT) family, which is evolutionarily widespread [10], or the nucleoside-H^+^ symporter (NHS) family, which is confined in bacteria [11]. The known gemcitabine transporters encoded in human fall in either the CNT (SLC28) or the equilibrative nucleoside transporter (ENT) family (SLC29) [12,13] and the transport of gemcitabine into pancreatic tumor cells has been primarily linked to hENT1 [14,15,16] and, to a minor extent, to hCNT1 or hCNT3 [17,18,19]. The functional knowledge of the bacterial gemcitabine transporters of the tumor microbiome is important to fully understand the involvement of the tumor-associated bacteria in the chemoresistance phenomenon and highlight the specificity differences between the bacterial and the human transporters.

In the present study, we investigate the complement of gemcitabine transporters of the CNT and NHS families in *E. coli* K-12 and two common gamma-proteobacteria species of the pancreatic tumor microbiome (*Klebsiella pneumoniae* and *Citrobacter freundii*). The results show that *E coli* K-12 contains two efficient high-affinity gemcitabine transporters with distinct specificity properties, namely, NupC and NupG; *C. freundii* has two gemcitabine transporters, which are orthologs and functionally equivalent to NupC and NupG, respectively; and *K. pneumoniae* has three gemcitabine transporters, of which two are orthologs and functionally equivalent to NupC and NupG, respectively, and the third is a functional variant of NupC, named KpNupC2. Our study reveals that these bacterial transporters have a higher affinity for gemcitabine than their human counterparts in tumor cells.

## 2. Results

### 2.1. NupC and NupG Are Efficient Gemcitabine Transporters of Escherichia coli K-12

The *E. coli* genome contains six members of the nucleoside transporter families CNT or NHS, namely, NupC, NupX, and PsuT/YeiM of the CNT family and NupG, XapB, and YegT of the NHS family. The available functional evidence is scant. NupC has been characterized as a pyrimidine-preferring nucleoside transporter [20] and NupG as a broad-specificity pyrimidine/purine nucleoside transporter [11], whereas PsuT/YeiM and XapB are referred to as pseudouridine and xanthosine transporters, respectively, based on their association with corresponding pseudouridine [21] and xanthosine utilization operons [22]; XapB has been proposed to be a xanthosine-preferring nucleoside transporter [22]. Concerning gemcitabine, only NupC has been pointed out to be relevant to gemcitabine transport based on earlier studies of the functional characterization of NupC through heterologous expression in *Xenopus laevis* oocytes [20] and the effect of NupC deletion mutants on the gemcitabine metabolism [8] or the development of *E. coli* resistance against gemcitabine [23].

**Figure 1:**
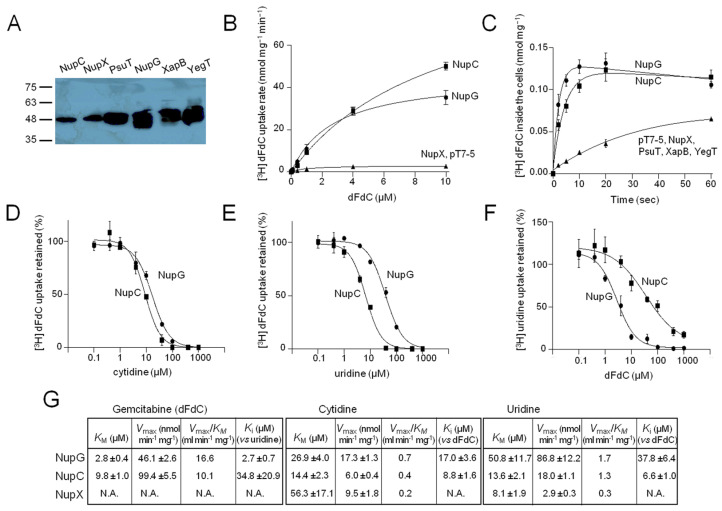
Gemcitabine-related transport properties of *E. coli* CNTs and NHSs. *E. coli* JW2389 expressing the indicated CNT or NHS homologs from pT7-5/-*BAD* were analyzed for protein levels in the membrane (**A**) and transport of [^3^H]-gemcitabine (**B**–**E**) or [^3^H]-uridine (**F**), as indicated. Panel (**A**): Membrane fractions (25 μg of membrane protein per lane) were subjected to SDS-PAGE (12%) and western blotting using HRP-conjugated streptavidin. Molecular mass standards (in kDa) were run in parallel, as indicated on the left. Panels (**B**,**C**): [^3^H]-Gemcitabine (dFdC) uptake was assayed at 0.1 μM ((**C**), time course) or at 5 s to measure the transport rate at a range of concentrations ((**B**), kinetics). Panels (**D**,**E**): Kinetics of inhibition of [^3^H]-gemcitabine (0.1 μM) uptake by cytidine (**D**) or uridine (**E**) based on measurements of the transport rate at 5 s. Panel (**F**): Kinetics of inhibition of [^3^H]-uridine (0.1 μM) uptake by gemcitabine based on measurements of the transport rate at 5 s. Panel (**G**): *K*_M_, *V*_max_, and *K*_i_ values deduced from experiments shown in (**B**–**E**) and in Supplementary Figure S1. The values presented in panels (**B**–**G**) are the means of 3–5 determinations with the SD. The values obtained with the vector alone were subtracted from the measurements in all cases (except in the time course shown in panel (**C**)); the values obtained with the vector alone (indicated as pT7-5) are also given in the kinetics of panel (**B**) for comparison.

Here, we examine the gemcitabine transporting potential of the six nucleoside-related gene products of *E. coli* K-12 after expressing the genes extrachromosomally in the genetic background of *Ε. coli* JW2389 (Δ*nupC*) (Figure 1A). We show that [^3^H]gemcitabine can be transported with high affinity (based on the *K*_M_ values) and efficiency (based on *V*_max_/*K*_M_ values) by NupC and NupG but not by NupX, PsuT, XapB, or YegT (Figure 1B,C). Concerning the related pyrimidine nucleosides cytidine and uridine, we found that NupC, NupG, and NupX can transport both cytidine and uridine. However, NupX shows comparatively low efficiency (low *V*_max_/*K*_M_ values) for uridine and cytidine and low affinity (high *K*_M_ value) for cytidine (Supplementary Figure S1 and Figure 1G). The gemcitabine uptake activity of NupC or NupG is inhibited competitively by cytidine (Figure 1D) and uridine (Figure 1E); the same is true of the inhibition of NupC or NupG uridine uptake activity by gemcitabine (Figure 1F). Based on the calculated *K*_M_ and *K*_i_ values, NupG exhibits 3.5-fold higher affinity (3.5-fold lower *K*_M_) for gemcitabine than NupC but approximately 2-fold lower affinity for cytidine and 4- to 6-fold lower affinity for uridine. NupC, on the other hand, exhibits similar *K*_M_ or *K*_i_ values for uridine and cytidine, and a *K*_M_ for gemcitabine that is only 1.5- to 1.8-fold lower than the ones for cytidine and uridine (Figure 1G). The *Κ*_Μ_ values determined for uridine or gemcitabine uptake by NupC and for uridine uptake by NupG are in the same range as the ones reported previously for NupC [20] and NupG [24].

A specificity profile analysis of NupC and NupG using assays of [^3^H]-uridine (0.1 μM) uptake and [^3^H]-gemcitabine (0.1 μM) uptake in the presence of 10^3^- to 10^4^-fold molar excess of unlabeled nucleosides shows that NupC is inhibited to completeness by all pyrimidine nucleosides (uridine, gemcitabine, cytidine, and thymidine) and adenosine but does not recognize guanosine, inosine, or xanthosine, whereas NupG is inhibited to completeness by all nucleosides tested except xanthosine (Supplementary Figure S2), in agreement with previous findings [24]. Of the other CNT and NHS homologs, XapB, which shares high sequence similarity with NupG and does not differ from NupG in the predicted binding-site region [11], exhibits a unique functional profile as it transports xanthosine. In addition, its transport activity is not inhibited to a substantial extent by any other nucleoside (Supplementary Figure S3), whereas xanthosine, uridine, or cytidine transport activity was not detected in our experiments for PsuT/YeiM or YegT.

### 2.2. Phylogenetic Analysis of NupC and NupG Homologs in Proteobacteria

We focused our analysis on the phylum of Proteobacteria because proteobacteria, especially gamma-proteobacteria, are enriched in the pancreatic tumor-associated microbiome relative to the gut microbiome and are common in pancreatic tumors [8,9]. We performed a comprehensive phylogenetic analysis of the families CNT and NHS in the phylum of Proteobacteria to identify major clades relevant to NupC and NupG, respectively, and assign other proteobacterial relatives of NupC and NupG to distinct phylogenetic clades. This strategy allowed us to elucidate multiple closely related homologs of the *E. coli* gemcitabine-transporting members, including homologs from two species commonly found in pancreatic tumor microbiomes (*K. pneumoniae* and *C. freundii*).

**Figure 2:**
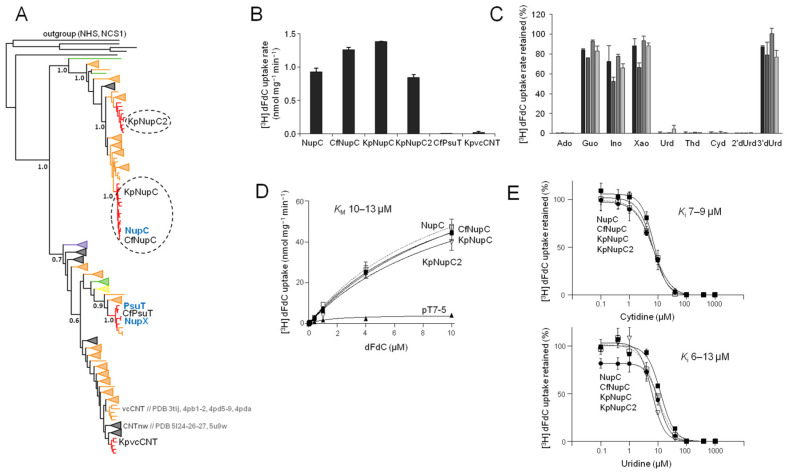
Phylogeny and gemcitabine transport properties of *C. freundii* and *K. pneumoniae* CNTs. Panel (**A**): Phylogenetic analysis of 275 CNT homologs representing one fully sequenced genome per genus for all proteobacteria, as retrieved from the IMG/M database at JGI (the complete phylogenetic tree is given in Supplementary Figure S4). The evolutionary history was inferred by the Maximum Likelihood method based on the Jones–Taylor–Thornton model as implemented in MEGA7. The tree with the highest log likelihood is shown. The percentage of trees in which the associated taxa were clustered together is shown (as decimal) next to the indicated major branches. The outgroup (shown on top) consists of *E. coli* members of families NHS (NupG, XapB, and YegT) and NCS1 (YbbW and CodB, appearing as a separate clade). Clades shown as cuneiforms consist of multiple homologs. Different colors indicate different classes of Proteobacteria, including alpha- (green), beta- (yellow), gamma- (red for Enterobacteriaceae and orange for all the others), delta- (purple), or epsilon- (dark purple) proteobacteria or clades with homologs from more than one class (gray). The homologs from *E. coli* K-12, *K. pneumoniae* ATCC 25955, and *C. freundii* ATCC 8090, as well as the structurally known homologs (with the corresponding PDB accession numbers), are indicated. Panel (**B**): [^3^H]-Gemcitabine (dFdC) transport rates (0.1 μΜ) by *E. coli* JW2389 expressing the indicated homologs from pT7-5/-*BAD*. Panel (**C**): Inhibition of [^3^H]-gemcitabine (0.1 μΜ) uptake rate of *E. coli* JW2389 expressing NupC, CfNupC, KpNupC, or KpNupC2 (darker to lighter gray in the histogram) by the indicated unlabeled nucleosides (0.1 mM). Panel (**D**): Kinetics of [^3^H]-gemcitabine transport. Panel (**E**): Dose–response inhibition curves of the [^3^H]-gemcitabine (0.1 μΜ) uptake rate by cytidine (above) and uridine (below). The data in (**D**,**E**) for NupC, CfNupC, KpNupC, and KpNupC2 are given as open rectangles (and interrupted lines), closed circles, closed rectangles, and open inverted triangles, respectively. The transport rates are deduced from measurements at 5 sec and given as the means of 3–5 determinations with the SD. The values obtained with the vector alone were subtracted from the measurements in all cases; the values obtained with the vector alone (indicated as pT7-5) are also given in the kinetics plot (**D**) for comparison.

The homologs from the CNT family comprise two major monophyletic groups, of which one contains NupC and the other contains the other two *E. coli* members (NupX and PsuT) and the two structurally known CNTs (vcCNT [10,25] and CNTnw [26]) in separate subclades (Figure 2A and Supplementary Figure S4). The homologs from *K. pneumoniae* are distributed in the NupC clade (two homologs) and the NupX/vcCNT clade (one homolog). Of them, one is closely related (ortholog) to NupC and was named KpNupC, the second one is a NupC paralog (72% identity) and was named KpNupC2, and the third one is distantly related to NupC (30% identity) but is most related to vcCNT (70% identity) and was named KpvcCNT (Figure 2A and Supplementary Figure S5). *C. freundii* has two homologs, one closely related to NupC and the other closely related to PsuT, that were named CfNupC and CfPsuT, respectively (Figure 2A).

**Figure 3:**
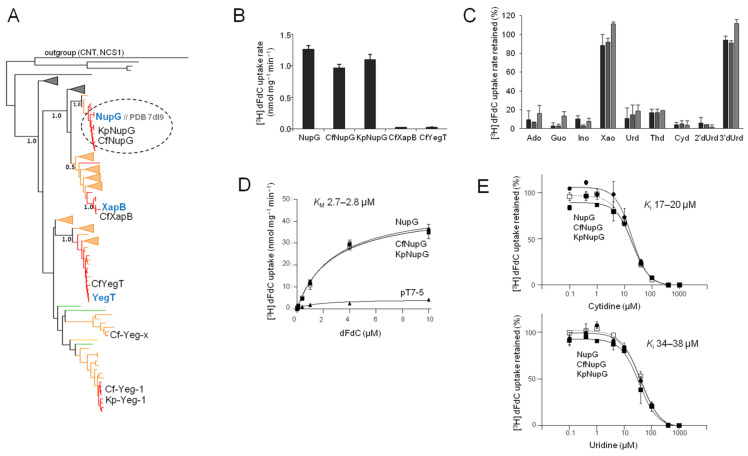
Phylogeny and gemcitabine transport properties of *C. freundii* and *K. pneumoniae* NHSs. Panel (**A**): Phylogenetic analysis of 146 NHS homologs representing one fully sequenced genome per genus for all proteobacteria as retrieved from the IMG/M database at JGI (the complete phylogenetic tree is given in Supplementary Figure S5). The outgroup (shown on top) consists of *E. coli* members of families CNT (NupC, NupX, and PsuT) and NCS1 (YbbW and CodB, appearing as a separate clade). Other methodological details are shown in Figure 2A. The homologs from *E. coli* K-12, *K. pneumoniae* ATCC 25955, and *C. freundii* ATCC 8090, as well as the PDB accession number of the structurally known NupG, are indicated. Panel (**B**): [^3^H]-Gemcitabine (dFdC) transport rates (0.1 μΜ) by *E. coli* JW2389 expressing the indicated homologs from pT7-5/-*BAD*. Panel (**C**): Inhibition of [^3^H]-gemcitabine (0.1 μΜ) uptake rate of *E. coli* JW2389 expressing NupG, CfNupG, or KpNupG (darker to lighter gray in the histogram) by the indicated unlabeled nucleosides (0.1 mM). Panel (**D**): Kinetics of [^3^H]-gemcitabine transport. Panel (**E**): Dose–response inhibition curves of the [^3^H]-gemcitabine (0.1 μΜ) uptake rate by cytidine (above) and uridine (below). The data in (**D**,**E**) for NupG, CfNupG, and KpNupG are given as open rectangles (and interrupted lines), closed circles, and closed rectangles, respectively. The transport rates are deduced from measurements at 5 sec and given as the means of 3–5 determinations with the SD. The values obtained with the vector alone were subtracted from the measurements in all cases; the values obtained with the vector alone (indicated as pT7-5) are also given in the kinetics plot (in (**B**)) for comparison.

The homologs from the NHS family comprise three major phylogenetic groups, one of which is clearly separated and contains NupG and XapB (Figure 3A and Supplementary Figure S6). *C. freundii* has five homologs, distributed in the NupG/XapB clade (two homologs) and the other two groups. Three of them are closely related (87–95% identical) to NupG, XapB, or YegT and were named CfNupG, CfXapB, and CfYegT, respectively. The remaining two homologs are in distantly related subclades but more related to YegT (36% identity) than to NupG or XapB and were named Cf-Yeg-1 and Cf-Yeg-x (Figure 3A; Supplementary Figure S7). Only two homologs were found in *K. pneumoniae*, one closely related to NupG and the other closely related to Cf-Yeg-1, and were named KpNupG and Kp-Yeg-1, respectively (Figure 3A).

### 2.3. Functional Characterization of the NupC Homologs of K. pneumoniae and C. freundii and the Identification of CfNupC, KpNupC, and KpNupC2 as Efficient Gemcitabine Transporters

Of 262 fully sequenced *K. pneumoniae* genomes (based on information available in the JGI IMG/M database [27], February 2022), 221 contain at least one CNT homolog; of them, 201 contain all three CNTs (KpNupC, KpNupC2, and KpvcCNT; Figure 2A) and the remaining 20 genomes contain either KpNupC alone (16 strains) or KpNupC and KpvcCNT (four strains). Of the 54 fully sequenced *C. freundii* genomes, 32 have at least one CNT homolog; 28 of them contain both CfNupC and CfPsuT, and four have CfNupC alone (Supplementary Table S1). We mobilized these five CNT genes from the genomes of *K. pneumoniae* ATCC 25955 and *C. freundii* ATCC 8090 accordingly and transferred them to pT7-5/-*BAD* plasmid vectors for expression in *Ε. coli* JW2389 (Δ*nupC*). After confirmation of expression in the *E. coli* plasma membrane (Supplementary Figure S8), we examined the relevant gene products for the transport of [^3^H]-gemcitabine in cell-based transport assays.

We found that CfNupC, KpNupC, and KpNupC2 transport [^3^H]-gemcitabine (0.1 μM) at rates comparable to NupC, whereas CfPsuT or KpvcCNT does not transport gemcitabine (Figure 2B).

Kinetic analysis shows that CfNupC, KpNupC, and KpNupC2 transport [^3^H]-gemcitabine with *K*_M_ (10–13 μΜ) and *V*_max_ values (87–102 nmol min^−1^ mg^−1^) that are essentially indistinguishable from those of NupC (Figure 2D; Table 1).

The inhibition profiles of [^3^H]-gemcitabine transport by other nucleosides show that, similar to NupC, the homologs CfNupC, KpNupC and KpNupC2 recognize all the natural pyrimidine nucleosides (including uridine, cytidine, and thymidine (i.e., 2′-deoxy-thymidine)), the analog 2′-deoxy-uridine (but not 3′-deoxy-uridine), and adenosine (but not any other purine nucleoside) with high affinity (Figure 2C); the *K*_i_ values of CfNupC, KpNupC, or KpNupC2 for uridine and cytidine are similar to the ones of NupC (Figure 2E; Table 1). In addition, the *K*_i_ values of KpNupC for 2′-deoxy-uridine, thymidine, or adenosine are very similar to the ones of NupC, whereas KpNupC2 differs from NupC in having a roughly twofold lower affinity for thymidine and 2′-deoxy-uridine (Table 1).

### 2.4. Functional Characterization of the NupG Homologs of K. pneumoniae and C. freundii and the Identification of CfNupG and KpNupG as Efficient High-Affinity Gemcitabine Transporters

Regarding the NHS family, of 262 fully sequenced *K. pneumoniae* genomes, 218 (83%) contain at least one NHS homolog. Of them, 197 contain two homologs (KpNup and Kp-Yeg-1) and the remaining 21 genomes contain only KpNupG. Of the 54 fully sequenced *C. freundii* genomes, 33 contain at least one NHS homolog, of which 9 contain five homologs (as designated in Figure 3A), 19 have four homologs (CfNupG, CfXapB, CfYegT, and Cf-Yeg-x), and 5 have three homologs (CfNupG, CfXapB, and CfYegT) (Supplementary Table S1). We mobilized the five most relevant NHS genes (except Cf-Yeg-x and Cf-Yeg-1/Kp-Yeg-1) from the genomes of *K. pneumoniae* ATCC 25955 and *C. freundii* ATCC 8090 accordingly and transferred them to pT7-5/-*BAD* plasmid vectors for expression in *Ε. coli* JW2389 (Δ*nupC*). After confirmation of expression in the *E. coli* plasma membrane (Supplementary Figure S8), we examined the relevant gene products for the transport of [^3^H]-gemcitabine in cell-based transport assays.

We found that CfNupG and KpNupG transport [^3^H]-gemcitabine (0.1 μM) at rates comparable to NupG, whereas CfXapB and CfYegT do not transport gemcitabine (Figure 3B); the kinetic analysis shows that CfNupG and KpNupG are essentially indistinguishable from NupG in the *K*_M_ (2.7–2.8 μΜ) and *V*_max_ (45–47 nmol min^−1^ mg^−1^) of [^3^H]-gemcitabine transport (Figure 3D; Table 1).

The inhibition profiles of [^3^H]-gemcitabine transport show that, similar to NupG, CfNupG and KpNupG recognize a wide range of nucleosides with high affinity, including pyrimidine nucleosides (uridine, cytidine, thymidine (i.e., 2′-deoxy-thymidine), and 2′-deoxy-uridine (but not 3′-deoxy-uridine)) and purine nucleosides (adenosine, guanosine, and inosine, but not xanthosine) (Figure 3C); the *K*_i_ values of CfNupG or KpNupG for uridine and cytidine are very similar to the ones of NupG (Figure 3E; Table 1). In addition, the *K*_i_ values of KpNupG for 2′-deoxy-uridine, thymidine, adenosine, guanosine, or inosine are indistinguishable from the ones of NupG (Table 1).

### 2.5. Distinction of the NupG Functional Profile from the NupC Functional Profile

As summarized in Table 1, all four studied NupC homologs from three different enterobacterial species exhibit the same gemcitabine-related profile (Figure 2C–E), distinct from the set of the three NupG homologs, which are also functionally equivalent to each other (Figure 3C–E). The NupG profile is characterized by a four- to fivefold higher affinity (lower *K*_M_) and twofold higher efficiency (*V*_max_/*K*_M_) for gemcitabine transport relative to NupCs (Figure 2D and Figure 3D; Table 1), the recognition of both pyrimidine and purine nucleosides, including guanosine and inosine, which are not ligands for NupCs (Figure 2C and Figure 3C), and a three- to fivefold lower affinity for adenosine (Table 1), three- to sixfold lower affinity for uridine (Figure 2E and Figure 3E), and two- to threefold lower affinity for thymidine and cytidine relative to NupCs (Figure 2E and Figure 3E, and Table 1). Among NupCs, KpNupC2, a paralog of KpNupC, deviates only by having a roughly twofold lower affinity for the 2′-deoxy nucleosides (2′-deoxy-uridine and thymidine) relative to the other NupCs (Figure 2 and Table 1).

## 3. Discussion

Proteobacteria are enriched in the bacterial populations colonizing pancreatic ductal adenocarcinoma tumors relative to the gut microbiome. Gamma-proteobacteria that are common in these microbiome populations contain a >800-nt long isoform of cytidine deaminase (CDD_L_), which has been correlated with the phenomenon of chemoresistance since it can rapidly convert gemcitabine into the less toxic 2**′**,2**′**-difluoro-2**′**-deoxyuridine (dFdU) [8]. On the other hand, the transmembrane transporters responsible for the uptake of the drug by the bacteria are not fully known. The import of gemcitabine into the bacterial cells has been associated with the nucleoside transporter NupC of the CNT family based on the fact that only NupC has been linked to gemcitabine transport in the gamma-proteobacterium *E. coli* K-12 model from earlier studies [20]. However, prior to the present study, no functional knowledge was available for the potential gemcitabine transport systems in *K. pneumoniae*, *C. freundii,* or any other gamma-proteobacteria, and even in *E. coli*, complete knowledge of the potential gemcitabine transporters was missing. In this work, we shift attention to a more systematic investigation of the potential bacterial transporters of the drug. We show that *E. coli* K-12 contains two high-affinity gemcitabine transporters, of which one (NupC) was known but not studied systematically in this respect in the past and the other (NupG of the NHS family) is shown here as a gemcitabine transporter for the first time. We also show that both NupC and NupG are present as gemcitabine transporters in two related Enterobacteriaceae species, i.e., *K. pneumoniae* and *C. freundii*, which are common and possibly linked to chemoresistance in pancreatic tumor microbiomes [8,9].

Our transport kinetic analysis shows that both NupC and NupG have higher kinetic affinities for gemcitabine relative to their human counterparts, i.e., *K*_M_ of 2–3 μΜ (NupG) or 10–13 μΜ (NupC), compared to 0.2–0.3 mM for hENT1 [28,29,30] and 20–60 μM for hCNT1 or hCNT3 [28,29,30,31,32]. The main transporter shown to mediate gemcitabine uptake in human cell lines is hENT1 (SLC29A1), and clinical correlation studies have shown that low expression of hENT1 in pancreatic adenocarcinoma is linked with poor outcomes of gemcitabine treatment [14,15,16]. NupG might be an interesting new candidate for further research of its substrate specificity in comparison to hENT1. Unlike NupC, which is a member of the evolutionarily widespread CNT family, NupG belongs to the prokaryote-specific NHS family, which is structurally distinct from the nucleoside transporter families (CNT and ENT) in humans. NHS and ENT are distantly related, both belonging to the Major Facilitator Superfamily [33] sharing the same overall fold and mechanistic motif (rocker-switch mechanism) [34]. However, NupG (NHS) and hENT1 are unrelated in sequence, have different binding site residues, and differ in their functional properties. hENT1 is a uniporter, NupG is a proton symporter, and hENT1 has a 100-fold lower affinity for gemcitabine (see above) and at least 10-fold lower affinity for uridine [35,36]; both proteins show broad specificity for purine and pyrimidine nucleosides, but hENT1 can additionally transport nucleobases with roughly 10-fold lower affinities [37]. hENT1 has been studied for recognition of a range of antimetabolite nucleoside analogs and has been shown to have a high affinity for several of them (dideoxycytidine, dideoxythymidine, azidothymidine, ribavirin, dideoxyinosine, cladribine, and dipyridamole) [35,36,37,38,39]. It would be interesting to assay NupG for recognition of these and other similar analogs to elucidate potential hENT1 substrate motifs that are not recognized by NupG.

Another intriguing feature with respect to NupG is derived from the comparison of the binding pocket residues in its recently solved structure [11] to other members of the NHS family. The ribose moiety of the nucleoside (uridine) in the structure of NupG is stabilized with hydrogen bonds from three conserved residues (R136, T140, and E264), which are invariable in the proteobacterial NHS transporters. The same is true of the neighboring D323, which is not directly in contact with the substrate but is considered crucial for coupling substrate binding with protonation based on the properties of the D323A and D323N mutants [11]. However, additional residues that interact with the nucleobase moiety through hydrogen bonds (Q225, N228, Q261, and Y318) or π–π interactions (F322 and F143) are also invariable in the monophyletic cluster containing the NupG and XapB homologs (see Figures S6 and S7). In contrast to NupG, which is of broad specificity but does not transport or recognize xanthosine (Figure S2) [11], XapB appears to be selective for xanthosine transport (Figure S3) [22]. Thus, it follows that residues at the periphery of the binding pocket are crucial for the substrate profile of NupG and underlie the functional distinction between NupG and XapB.

Another aspect of our work concerns the phylogenetic analysis of the distribution of NupC, NupG, and related transporter homologs among proteobacteria. This is important to investigate to understand the realm of functional transporters that might be relevant to the chemoresistance-related metabolism of gemcitabine in tumor microbiomes. Through a summary of our key observations, we have found that NupC orthologs (constituting a subgroup of the NupC clade, sharing 74–99% pairwise sequence identity) are clustered in almost all families of Enterobacterales, whereas the NupG orthologs (a subgroup of the NupG/XapB clade, with 78–99% pairwise sequence identity) are mostly confined in Enterobacteriaceae (Supplementary Table S2). All species containing NupC and/or NupG possess the long cytidine deaminase isoform CDD_L_, and the few species of Enterobacterales lacking CDD are devoid of both NupC and NupG (Table S2). In addition to NupC, the paralog NupC2 (which is probably a gemcitabine transporter similar to NupC based on the results of KpNupC2) is also present in several species, including most Enterobacteriaceae. Interestingly, although most *K. pneumoniae* strains contain all three transporters, i.e., NupC, NupC2 and NupG, 13% of them lack NupC2, 6% contain only NupC, and 5% contain only NupG as a potential gemcitabine transporter (Table S1). Of the other CNTs or NHSs, which show no detectable gemcitabine transport (Figure 1, Figure 2 and Figure 3), KpvcCNT is related in sequence and phylogeny (Figure S6) to vcCNT, which has been characterized as a uridine transporter with high affinity for uridine and cytidine, but very low affinity for gemcitabine (*K*_D_ of about 1.5 mM, which is 40-fold higher than that of uridine, based on fluorescence anisotropy measurements) [25]. The presence of KpvcCNT orthologs is not correlated with genomes containing the active CDD_L_ isoform in any of the Enterobacterales families (Table S2). Overall, the data imply that either NupC or NupG or both NupC and NupG might be involved in the cellular uptake of gemcitabine depending on the enterobacterial family, strain, or species.

Apart from Enterobacterales, other bacteria also contain the CDD_L_ isoform that has been implicated in gemcitabine resistance [8]. In the analysis of bacterial species from the Kyoto Encyclopedia of Genes and Genomes (KEGG) [40] shown in [8] (Table S5 in [8]), 98.4% of the genomes containing CDD_L_ are gamma-proteobacteria. Enterobacterales constitute two-thirds of these genomes. The remaining one-third of the genomes belong to genera that appear in our phylogenetic analysis of nucleoside transporters in clusters that are closely related to CNTnw [26] (*Haemophilus*, *Mannheimia*, *Aggregatibacter*, and *Pasteurella*) or vcCNT [10] (*Vibrio*, *Allivibrio*, *Aeromonas*, and *Shewanella*) (Figure S6). It seems plausible to assume that some of these homologs might be involved in the uptake of gemcitabine in the aforementioned gamma-proteobacteria.

Experimental evidence of *E. coli* indicates the association of NupC with gemcitabine transport through the properties of *nupC*-knockout mutants. One piece of this evidence refers to the partial abrogation of gemcitabine metabolism in CDD_L_-containing *E. coli* K-12 that lack the *nupC* gene, as observed from the compromised alleviation of the gemcitabine effect on a human pancreatic adenocarcinoma cell line [8]. This effect of *nupC* knockout is partial and much less pronounced (10-fold higher EC_50_) than the effect of CDD_L_ knockout (Figure 2C in [8]), implying the involvement of additional gemcitabine transporters. The second piece of evidence comes from the study of the adaptation of *E. coli* to gemcitabine through an experimental evolution strategy highlighting that *nupC* loss-of-function mutations are correlated with gemcitabine resistance [23]. In the context of this study, the authors also performed a genome-wide screen showing that several different single gene losses can confer resistance and impact bacterial drug degradation. Apart from NupC, other transporters, as well as metabolic genes and transcription factors, were among single-gene knockouts yielding gemcitabine resistance [23]. Some of the resistance effects are complex as they involve both increased import and increased deamination of gemcitabine. This is the case with *cytR* knockout since CytR is a repressor of numerous genes, including the genes for both gemcitabine transporters *nupC* and *nupG* and the gemcitabine deaminating enzyme *cdd*. Overall, it appears that multiple alternative mutation routes in *E. coli* could lead to gemcitabine chemoresistance.

In conclusion, the initial characterization of gemcitabine-related transporter properties of NupG in *E. coli*, *C. freundii*, and *K. pneumoniae* and the phylogenetic analysis of NupG, NupC, and related nucleoside transporters in the NHS and CNT families might broaden our understanding of the bacterial gemcitabine transporters involved in the phenomenon of bacteria-mediated chemoresistance and encourage experimentation to determine the differences between bacterial and human drug transporters at the molecular level. The experiments in this study demonstrate the existence of multiple high-affinity gemcitabine transporters in enterobacteria that might limit the availability of the drug within aggressive pancreatic tumors and inhibit gemcitabine treatment. Further experiments using co-cultures of pancreatic cancer cell lines with selected bacterial strains in the presence of gemcitabine will be needed to show the impact of these bacterial gemcitabine transporters on the inhibition of the gemcitabine effect. In parallel, the analysis of the substrate specificity differences between the bacterial and the human gemcitabine transporters will open the way for the improvement of the chemotherapeutic schemes against aggressive pancreatic cancers by aiding the development of new nucleoside analogs that might be more specific to the cancer cell targets and less effective as substrates for uptake by the bacterial cells.

## 4. Materials and Methods

### 4.1. Phylogenetic Analysis of CNT and NHS Families in Proteobacteria

As of February 2022, we selected all genomes in the phylum Proteobacteria from the IMG/M database at JGI [27] with a genome status marked as ‘Finished’. A total of 6662 bacterial strains were recovered and classified according to class (alpha-, beta-, gamma-, delta-, and epsilon-proteobacteria). Using *E. coli* NupC or NupG as a query for the CNT or NHS family, respectively, we performed a BLAST-p search in each of the five classes and retrieved all homologous sequences (cutoff E value 1 × 10^−5^). We identified 6434 sequences belonging to the CNT family and 4560 belonging to the NHS family. The data size underwent an initial reduction to 927 CNT and 388 NHS sequences by selecting homologs from one strain per species (a strain containing the maximum number of homologs for each species). Subsequently, the data size was further reduced to 275 CNT and 146 NHS sequences by retaining homologs from one strain per genus (a strain containing the maximum number of homologs for each genus). Both sets of sequences were aligned with Muscle and subjected to Maximum Likelihood (ML) phylogenetic analysis with MEGA7 [41].

### 4.2. Materials for Wet-Lab Experiments and General Considerations

[^3^H]-gemcitabine ([cytosine-5-^3^H(N)]-gemcitabine) (20.1 Ci mmol^−1^), [5,6-^3^H]-uridine (30.0 Ci mmol^−1^), [5-^3^H]-cytidine (27.7 Ci mmol^−1^), and [8-^3^H]-xanthosine (17.1 Ci mmol^−1^) were obtained from Moravek Biochemicals (Brea, CA, USA). The non-radioactive nucleosides and analogs were obtained from Sigma-Aldrich (St. Louis, MO, USA). The nucleosides were prepared in dimethyl sulfoxide (DMSO). The cell cultures were performed in Luria-Bertani broth (LB) or M9 minimal media (M9) in aerobic conditions. For all incubations in liquid media, *E. coli* cells were grown in a shaking incubator at 220 r.p.m. at 37 °C. Oligodeoxynucleotides were synthesized from Eurofins Genomics, Ebersberg, Germany. High-fidelity DNA polymerase, restriction endonucleases, alkaline phosphatase, and T4 DNA ligase were obtained from Takara Clontech. Horseradish peroxidase (HRP)-conjugated streptavidin was obtained from Millipore. All the other reagents were of analytical grade and obtained from commercial sources.

### 4.3. Bacterial Strains, Coding Sequences, and Plasmids

Genomic DNA from *Klebsiella pneumoniae* DSM 4799 (ATCC 25955) and *Citrobacter freundii* DSM 30039 (ATCC 8090) were obtained from Eurofins Genomics and used for the mobilization of the relevant CNT and NHS sequences. *E. coli* T184 (*lacI^+^O^+^Z^–^Y^–^ (A)*, *prsL*, *met^–^*, *thr^–^*, *recA*, *hsdR/F’*, and *lacI^q^O^+^ZD^118^*) was used for the mobilization of *E. coli* CNT and NHS sequences. The genes mobilized correspond to the coding sequences of *E. coli* NupC (P0AFF2), NupX (P33021), YeiM/PsuT (P33024), NupG (P0AFF4), XapB (P45562), and YegT (P76417) (UniProt numbers given in parentheses), *K. pneumoniae* KpNupC (A0A2W0KM59), KpNupC2 (WP_002898911), KpvcCNT (WP_004146034), and KpNupG (WP_038806797), and *C. freundii* CfNupC (A0A336NW46), CfPsuT (D2TRJ2), CfNupG (A0A7D6VR53), CfXapB (A0A7W3D7V4), and CfYegT (A0A7D6VSQ9) (trEMBL or NCBI accession numbers in parentheses). Other sequences of NHS homologs shown in Figure 3A are Cf-Yeg-x (A0A0D7M2I3), Cf-Yeg-1 (A0A7W3HTK5), and Kp-Yeg-1 (J2XAI5). The sequence alignments in Supplementary Figures S5 and S7 were performed using Multalin [42].

For expression in *E. coli* K-12, the coding sequence of each gene was transferred to a previously described plasmid vector pT7-5, which included the DNA sequence of the biotin-acceptor domain (*BAD*) of the oxaloacetate decarboxylase from *K. pneumoniae* as an insert between the *Apa*I and *Hin*dIII sites. This vector was designated pT7-5/-*BAD*. After insertion of the coding sequence at the appropriate orientation and frame, the resulting constructs contained the BAD sequence as a C-terminal tag of each CNT or NHS. Following expression, the tagged gene products were biotinylated in vivo during bacterial growth and allowed monitoring of the protein level in the *E. coli* membrane by western blotting [43].

*E. coli* BW25113 strains with appropriate transporter-gene single knockout (Keio collection) [44] (provided by the Coli Genetic Stock Center Culture Collection, through Horizon Discovery, Cambridge, UK) were used for the expression of the pT7-5/-*BAD*-borne CNT or NHS gene from the *lacZ* promoter/operator and transport assays. In particular, *E. coli* JW2389 (*NupC* knockout) was used for assays of gemcitabine, uridine, and cytidine transport, and *E. coli* JW2397 (*xapB* knockout) was used for assays of xanthosine transport. All *E. coli* strains were transformed according to Inoue et al. [45].

### 4.4. Molecular Cloning and Bacterial Growth

The coding sequences of the genes were amplified with PCR on the template of genomic DNA and transferred to pT7-5/-*BAD* by restriction fragment replacement between the *Bam*HI and *Apa*I sites. The sequences of synthetic oligodeoxynucleotides used as PCR primers are given in Supplementary Table S3. The coding sequence of all the constructs was verified by double-strand DNA sequencing (Eurofins Genomics, Ebersberg, Germany).

*E. coli* JW2389 or JW2397 harboring the pT7-5/-*BAD* based plasmids were grown aerobically at 37 °C in LB containing kanamycin (0.025 mg/mL) and ampicillin (0.1 mg/mL). Fully grown cultures (1 mL) of *E. coli* JW2389 were transferred to M9 (supplemented with 22.2 mM glucose as a C source and 20 mM NH_4_Cl as an N source), diluted 10-fold, allowed to grow to a mid-logarithmic phase (OD_600nm_ 0.5–0.6), induced with isopropyl-*β*-D-1-thiogalactopyranoside (IPTG) (0.5 mM) for 105 min at 37 °C, and harvested for use in transport assays or western blotting. Fully grown cultures (1 mL) of *E. coli* JW2397 were diluted 10-fold in LB, induced with IPTG as above, and harvested for use in the xanthosine transport assays.

### 4.5. Western Blot Analysis

*E. coli* JW2397 was washed twice in Tris-HCl (0.05 M), with a pH of 8.0, containing NaCl (0.1 M) and Na_2_EDTA (1 mM), supplemented with 4-(2-aminoethyl) benzenesulfonyl fluoride hydrochloride (AEBSF) (0.2 mM), and was used to prepare membrane fractions by osmotic shock, treatment with EDTA/lysozyme, and sonication [43]. Membrane fractions prepared from 10 mL cell cultures were harvested by ultracentrifugation in an Optima MAX-XP Ultracentrifuge (Beckman Coulter, Brea, CA, USA), normalized to a protein concentration of 100 μg per 50 μL in sample loading buffer, and subjected to SDS-PAGE (12%) (25 μg protein per lane). After electrophoresis, the proteins were electroblotted to a polyvinylidene difluoride membrane (Parablot PVDF; Macherey Nagel, Düren, Germany), and the BAD-tagged proteins were probed with HRP-conjugated streptavidin, which was used at a dilution of 1:50,000. Signals were developed with enhanced chemiluminescence (ECL).

### 4.6. Transport Assays and Kinetic Analysis

*E. coli* JW2389 were washed twice in MK buffer (MES 5 mM, with a pH of 6.5, containing 0.15 M KCl), normalized to an OD_420nm_ of 10.0 (corresponding to 35 μg of total protein per 50 μL) in the same buffer and assayed for the transport of [^3^H]-gemcitabine, [^3^H]-cytidine, or [^3^H]-uridine. Before initiating the transport reaction, the cells were energized by the addition of glycerol to a final concentration of 20 mM and equilibrated in the assay buffer for 3 min at 25 °C [46]. *E. coli* JW2397 was prepared and assayed for [^3^H]-xanthosine transport in KPi (0.1 M and pH of 7.5). All the transport reactions were performed at 25 °C. After the termination of reactions, the samples were rapidly filtered through Whatman GF/C filters, washed twice immediately with 3 mL of ice-cold KL buffer (KPi: 0.1 M, pH: 5.5, and LiCl: 0.1 M), and prepared for liquid scintillation counting.

To determine the *K*_M_ and *V*_max_ values, the data were fitted to the Michaelis–Menten equation using Prism 8. To obtain IC_50_ values in competitive inhibition experiments, the data were fitted to the equation *y* = B + (T − B)/(1 + 10 ^((log IC50 − log *x*) *h*)^) for sigmoidal dose–response (variable slope) using *Prism*8, where *x* is the concentration variable, *y* (the transport rate) ranges from T (top) to B (bottom), and *h* is the Hill coefficient. The *h* value was consistently close to −1, indicating competition for a single binding site. *K*_i_ values were calculated from the IC_50_ values based on the equation *K*_i_ = IC_50_/[1 + (S/*K*_M_)] (where S is the concentration of the radiolabeled substrate used and *K*_M_ is the corresponding value obtained for the relevant transporter in the kinetics assay) [47].

## Figures and Tables

**Table 1:** Kinetics and specificity of [^3^H]-gemcitabine transport.

Homolog	Kinetics of Transport			Inhibition by Other Nucleosides						
	*K*_M_ (μM)	*V*_max_ (nmol min^−1^ mg^−1^)	*V*_max_/*K_Μ_* (ml min^−1^ mg^−1^)	*K*_i_ (μM) (Cytidine)	*K*_i_ (μM) (Uridine)	*K*_i_ (μM) (Thymidine)	*K*_i_ (μM) (Adenosine)	*K*_i_ (μM) (Guanosine)	*K*_i_ (μM) (Inosine)	*K*_i_ (μM) (2′dUrd)
NupC	9.8 ± 1.0	99.4 ± 5.5	10.1	8.8 ± 1.6	6.6 ± 1.0	11.6 ± 3.9	5.4 ± 1.4	N.I.	N.I.	10.9 ± 6.5
CfNupC	12.8 ± 1.3	101.6 ± 6.3	7.8	7.2 ± 1.4	12.7 ± 3.0	inhibited	inhibited	N.I.	N.I.	inhibited
KpNupC	10.9 ± 2.0	92.8 ± 10.2	8.6	6.7 ± 1.3	10.8 ± 2.7	13.9 ± 3.0	7.7 ± 0.8	N.I.	N.I.	11.4 ± 1.6
KpNupC2	11.6 ± 3.2	87.3 ± 14.4	7.5	6.5 ± 1.0	6.1 ± 1.0	18.0 ± 3.9	7.9 ± 0.6	N.I.	N.I.	25.4 ± 8.3
NupG	2.8 ± 0.4	46.1 ± 2.6	16.6	17.0 ± 3.6	37.8 ± 6.4	32.6 ± 12.0	24.5 ± 9.3	15.3 ± 5.8	15.7 ± 6.0	20.2 ± 3.3
CfNupG	2.7 ± 0.3	47.4 ± 1.8	17.2	20.2 ± 3.8	33.6 ± 7.8	inhibited	inhibited	inhibited	inhibited	inhibited
KpNupG	2.8 ± 0.3	45.6 ± 1.8	17.1	18.1 ± 4.0	37.5 ± 8.5	32.1 ± 8.2	22.9 ± 5.4	18.3 ± 4.5	18.6 ± 7.8	19.0 ± 2.6

Data obtained from the kinetics of [^3^H]-gemcitabine (0.01–10 μM) transport and inhibition of [^3^H]-gemcitabine (0.1 μM) transport by the indicated non-labeled nucleosides (0.1–1000 μΜ) based on rate measurements at 5 sec with *E. coli* JW2389 expressing the corresponding constructs. N.I. indicates no inhibition in any of the concentrations tested. Inhibited: transport activity inhibited to completeness by 1000-fold excess (0.1 mM) of the indicated nucleoside.

## Data Availability

The original contributions presented in this study are included in the article and Supplementary Material. Further inquiries can be directed to the corresponding author.

## Supplementary Materials

The following supporting information can be downloaded at: https://www.mdpi.com/article/10.3390/ijms25137012/s1.
