# Supplementary material for: Elucidation of the Gemcitabine Transporters of Escherichia coli K-12 and Gamma-Proteobacteria Linked to Gemcitabine-Related Chemoresistance
Source: Int J Mol Sci. 2024 Jun 27;25(13):7012. doi: 10.3390/ijms25137012 (PMC11241209; doi:10.3390/ijms25137012)
Supplement: Supplementary file 1 [file ijms-25-07012-s001.zip › 1. Supplementary material to IJMS-Frillingos K-12.pdf]

Supplementary materials for the article

Elucidation of the gemcitabine transporters of *Escherichia coli* K-12 and gamma-proteobacteria linked to gemcitabine-related chemoresistance

Nikoleta Iosifidou, Eleni Anagnostopoulou, Maria Botou, Eirini Kalfa, Ekaterini Tatsaki, and Stathis Frillingos

This pdf file includes:

Figures S1 to S8

Captions for Tables S1 and S2

Table S3

Other supplementary materials for this manuscript include the following:

Table S1 (excel file, provided separately)

Table S2 (excel file, provided separately)

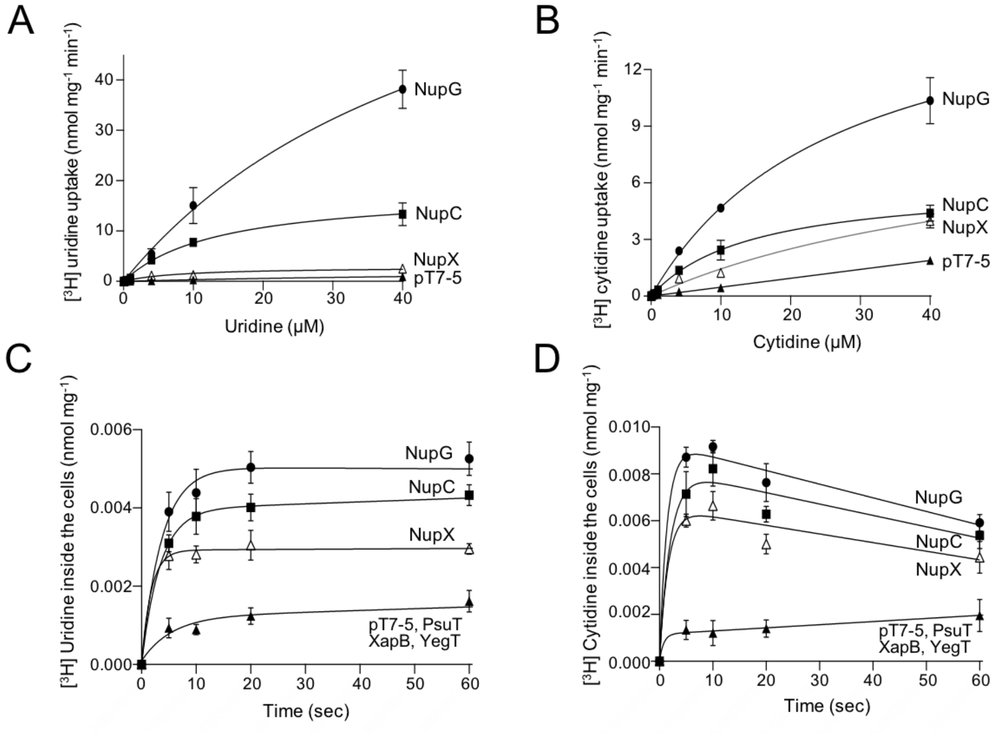

Figure S1: Uridine and cytidine transport

Kinetics of [^{3}H]-uridine (A) or [^{3}H]-cytidine uptake (B), based on measurements of transport rates at 5 sec, and time courses of [^{3}H]-uridine (0.1 µM) (C) and [^{3}H]-cytidine (0.1 µM) (D) uptake by *E. coli* JW2389 expressing the indicated CNT or NHS from pT7-5/-*BAD* vector. Each value given represents the means of three determinations with SD shown.

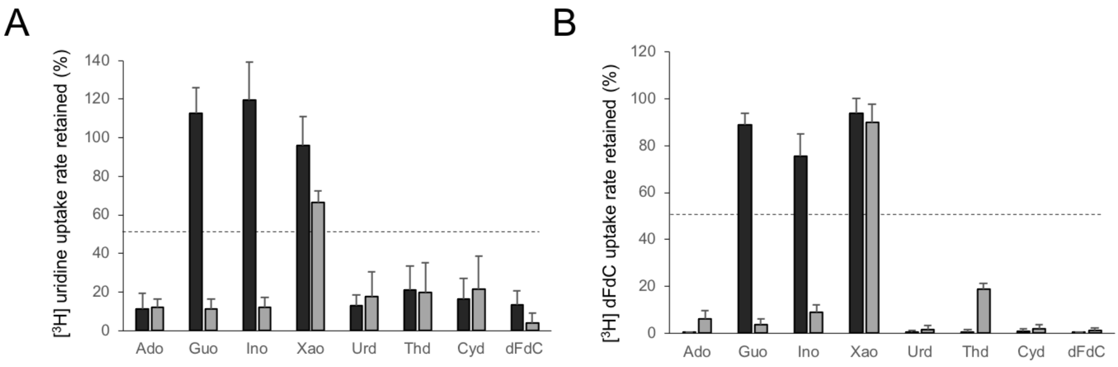

Figure S2: Specificity profiles of NupC and NupG.

Inhibition of [^{3}H]-uridine (0.1 µM) uptake rate of *E. coli* JW2389 expressing NupC (black) or NupG (gray) by unlabeled nucleosides (1 mM) (A); inhibition of [^{3}H]-gemcitabine (0.1 µM) uptake rate of *E. coli* JW2389 expressing NupC (black) or NupG (gray) by unlabeled nucleosides (0.1 mM) (B). Uptake rates were measured at 5 sec. Values obtained with vector pT7-5/-*BAD* alone were subtracted from the measurements in all cases. Each bar represents the means of 3-5 determinations with SD shown.

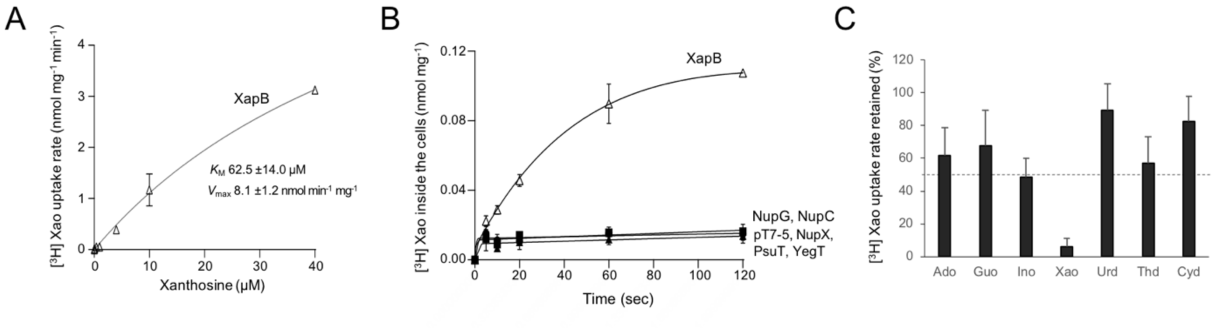

Figure S3: Xanthosine transport by XapB.

Kinetics of [^{3}H]-xanthosine uptake by *E. coli* JW2397 expressing XapB from pT7-5/-*BAD* vector (A); time course of [^{3}H]-xanthosine (1 µM) uptake by *E. coli* JW2397 expressing XapB compared to other NHS and CNT transporters, as indicated (B); inhibition of [^{3}H]-xanthosine (1 µM) uptake rate of *E. coli* JW2397 expressing XapB by unlabeled nucleosides (1 mM) (C). Uptake rates were measured at 10 sec (left panel) or 20 sec (right panel). Values obtained with vector pT7-5/-*BAD* alone were subtracted from the measurements in all cases (except in the middle panel). Each value given represents the means of 3-5 determinations with SD shown.

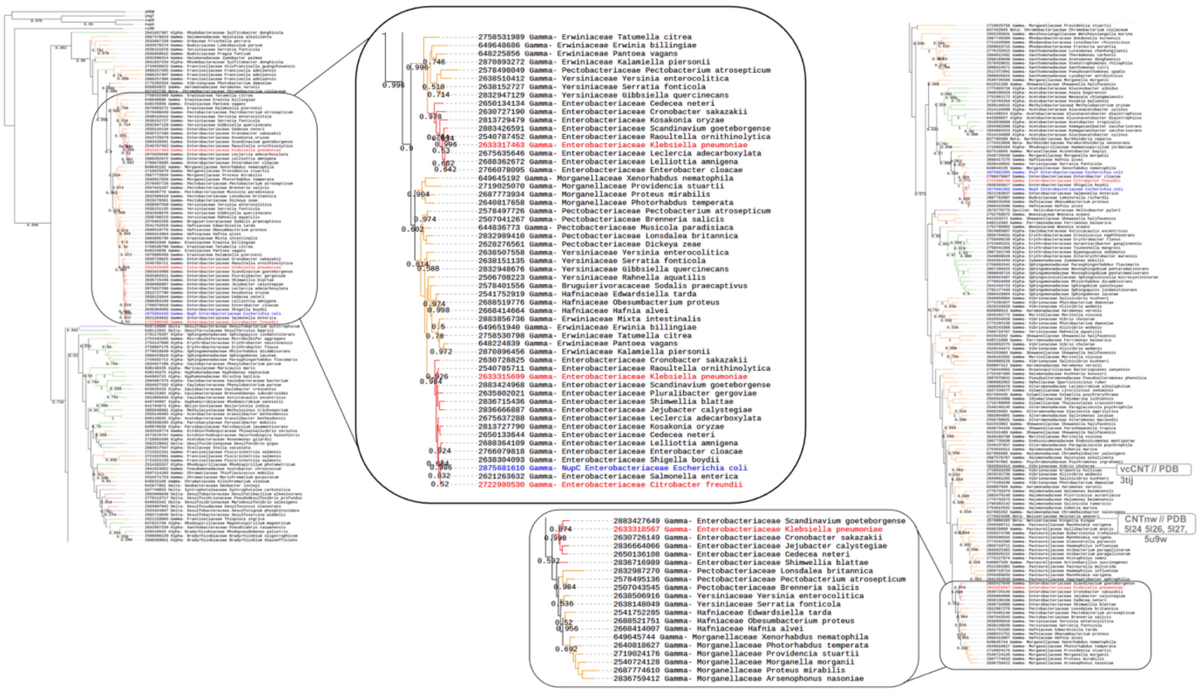

Figure S4: Phylogenetic analysis of CNT homologs.

Phylogenetic analysis of 275 CNT homologs representing one fully sequenced genome per genus for all Proteobacteria. Details are given in the legend to Fig. 2A. The clusters containing NupC and KpvcCNT, respectively, are shown enlarged.

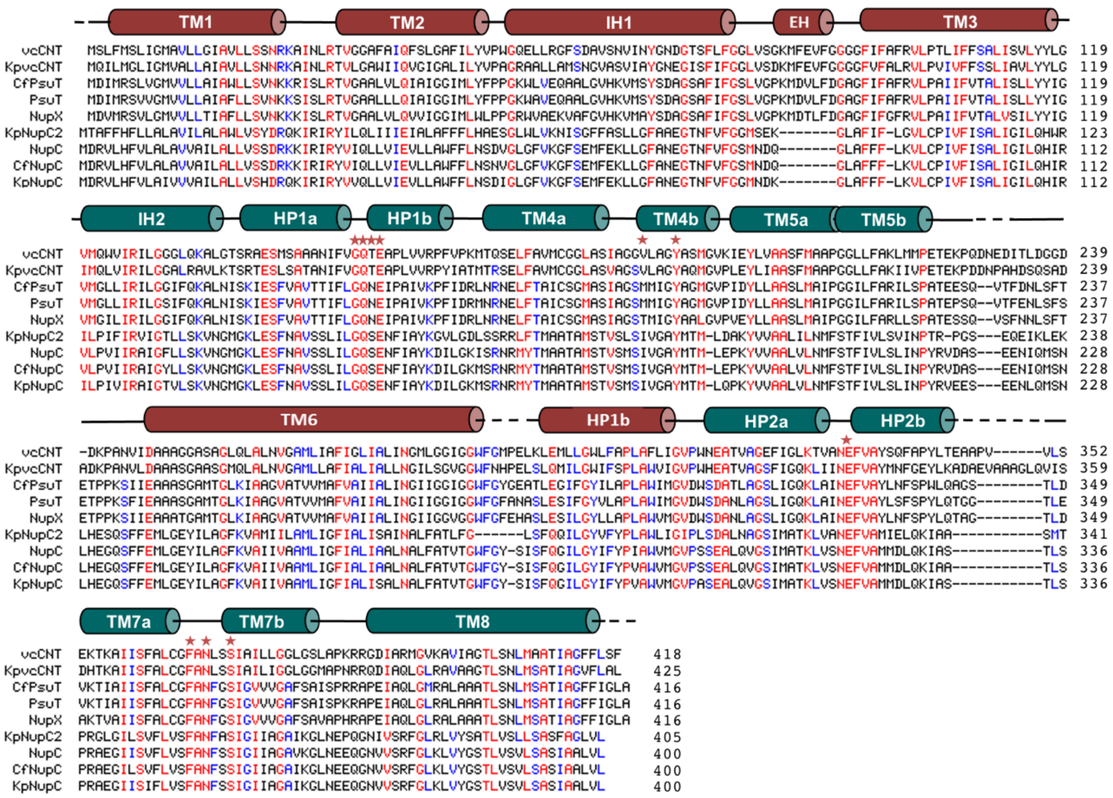

Figure S5: Sequence alignment of CNT homologs.

The indicated sequences were aligned using Multalin [Corpet, F. Multiple sequence alignment with hierarchical clustering. *Nucleic Acids Res.* **1988**, *16*, 10881-10890]. High consensus sites are shown in red and low consensus sites in blue. The positions and names of alpha-helices shown on top and important residues of the substrate binding region (indicated with asterisks) are according to vcCNT structure PDB 3tij [Johnson, Z. L., *et al*. *Nature* **2012**, *483*, 973-980]. The N-terminal 11-amino acid sequence of KpNupC2 has been omitted for clarity.

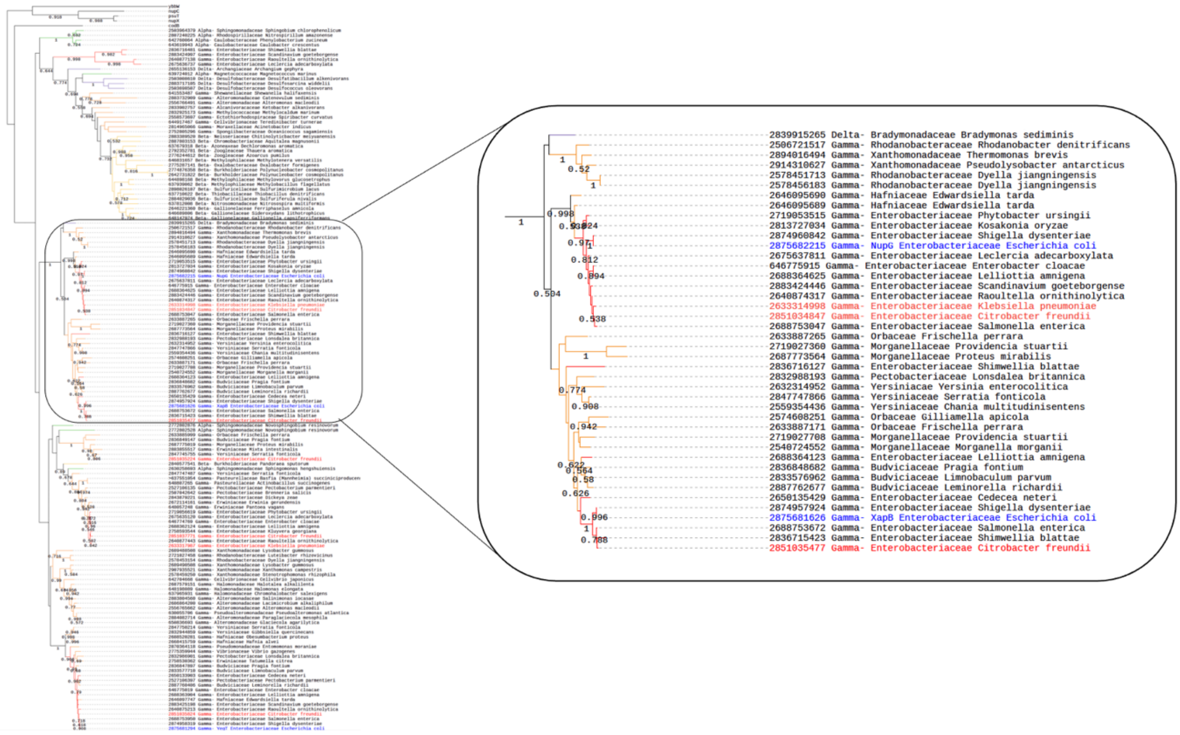

Figure S6: Phylogenetic analysis of NHS homologs.

Phylogenetic analysis of 146 NHS homologs representing one fully sequenced genome per genus for all Proteobacteria. Details are given in the legend to Fig. 3A. The cluster containing NupG is shown enlarged.

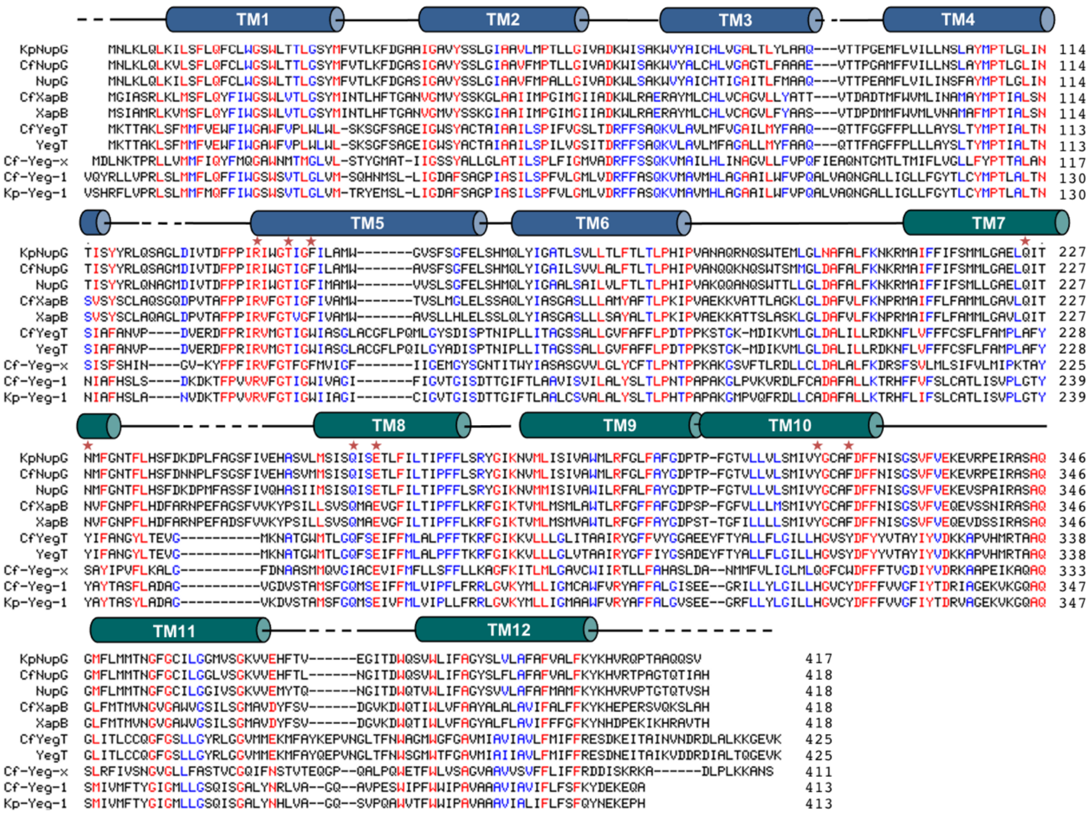

Figure S7: Sequence alignment of NHS homologs.

The indicated sequences were aligned using Multalin [Corpet, F. Multiple sequence alignment with hierarchical clustering. *Nucleic Acids Res.* **1988**, *16*, 10881-10890]. High consensus sites are shown in red and low consensus sites in blue. The positions and names of alpha-helices shown on top and important residues of the substrate binding region (indicated with asterisks) are according to NupG structure PDB 7dl9 [Wang, C., *et al. J. Biol. Chem.* **2021**, *296*: 100479]. The N-terminal 12-amino acid sequence of Cf-Yeg-1 and Kp-Yeg-1 has been omitted for clarity.

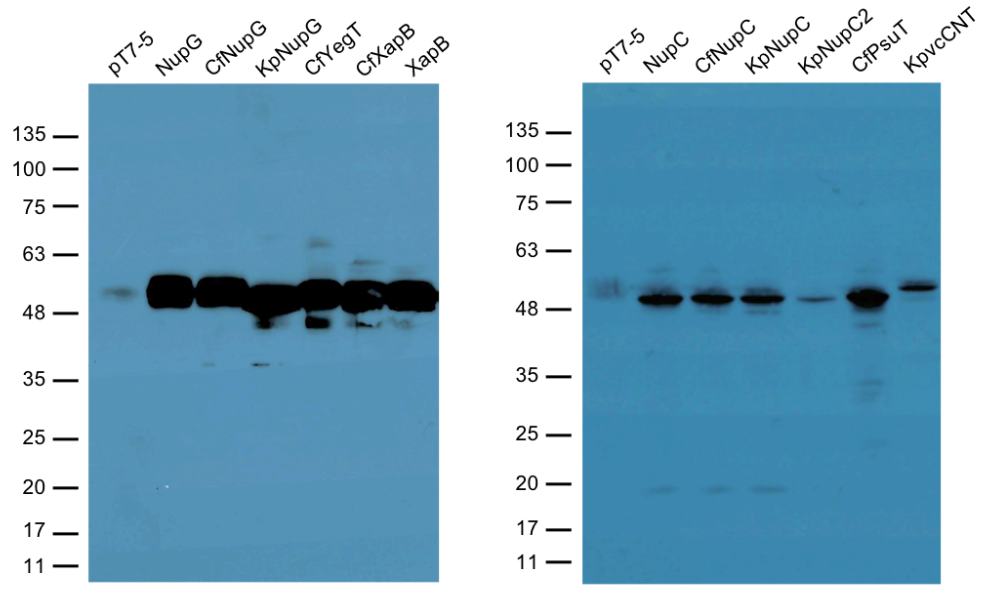

Figure S8: Protein products of CNT and NHS expressed in the *E. coli* membrane.

Membrane fractions prepared from *E. coli* JW2389 expressing the indicated CNT or NHS from pT7-5/-*BAD* vector were subjected to SDS-PAGE (12%) and western blotting using HRP-conjugated streptavidin. Each lane contains 25 μg of total membrane protein. Molecular mass standards (ranging from 135 kDa to 11 kDa) were run in parallel, as indicated on the left.

Table S1: CNT and NHS content of *K. pneumoniae* and *C. freundii*.

Designations of the homologs are based on their phylogenetic distribution.

Table S2: CDD, NupC and NupG in Enterobacterales.

NF stands for Not Found. Color code: CNT (light green), NHS (blue). CDD length is color coded with shades of dark green. Most genomes in each species have the same CDD length as shown, but *Proteus mirabilis* or *Providencia stuartii* include some genomes with a 894-nt long CDD as well. Designations of the CNT and NHS homologs are based on their phylogenetic distribution.

Table S3: Oligodeoxynucleotide primers used for transfer of genes to pT7-5/-*BAD*

Gene	Sense primer	Antisense primer
nupC	GATTCA**GGATCC**ATG/GAC/CGC/GTC/CTT/CAT/TTT/GTA/C	GATCAT**GGGCC/C**AG/CAC/CAG/TGC/TGC/GAT/TGA/CGC
nupX	GATTCA**GGATCC**ATG/GAT/GTC/ATG/AGA/AGT/GTT/CTG/G	GATAAT**GGGCC/C**GC/TAA/ACC/AAT/AAA/GAA/CCC/GGC/AAT/G
psuT	GACGCA**GGATCC**ATG/GAT/ATA/ATG/AGA/AGT/GTT/GTG	GATAAT**GGGCC/C**GC/CAG/ACC/AAT/AAA/GAA/TCC/TGC/AAT/AGT/C
nupG	GAGCAT**GGATCC**ATG/AAT/CTT/AAG/CTG/CAG/CTG/AAA/ATC/C	GATAAT**GGGCC/C**TG/GCT/AAC/CGT/CTG/TGT/GCC/TG
xapB	GATCAT**GGATCC**ATG/AGC/ATC/GCG/ATG/CGC/TTA/AAG/G	GATCAT**GGGCC/C**TG/AGT/CAC/CGC/TCG/ATG/CTT/TAT/C
ygeT	GATTCA**GGATCC**ATG/AAA/ACA/ACA/GCA/AAG/CTG/TC	GATTAT**GGGCC/C**TT/AAC/TTC/CCC/TTG/TGT/CAA/CG
CfnupC	GATCA**GGATCC**ATG/GAC/CGC/GTC/CTT/CAT/TTT/GTA/TTG	GATTAT**GGGCC/C**AG/CAC/TAG/TGC/TGC/GAT/AGA/TG
CfpsuT	GAGTCA**GGATCC**ATG/GAT/ATA/ATG/AGA/AGT/GTT/GTG/G	GATTAT**GGGCC/C**GC/CAG/TCC/GAT/AAA/GAA/ACC/GG
CfnupG	GAGTCA**GGATCC**ATG/AAC/CTT/AAG/CTG/CAG/CTG/AAA/G	GATTAT**GGGCC/C**TG/CCG/CGA/TAG/TTT/GGG/TGC/CTG
CfxapB	GGAGGT**GGATCC**ATG/GGT/ATC/GCA/TCT/CGC/TTA/AAG	CTATAA**GGGCC/C**TG/TGC/CAG/TGA/TTT/CTG/CGC/CCA/GTC/GTT/C
CfyegT	GATCAT**GGATCC**ATG/AAA/ACT/ACA/GTT/AAG/CTG/TCC/GTT/C	GATCAT**GGGCC/C**TT/TAC/TTC/CCC/TTG/TTT/CAA/CGC
KpnupC	GATTCA**GGATCC**ATG/GAC/CGC/GTC/TTG/CAT/TTT/GTC	GATTAT**GGGCC/C**AG/AAC/CAG/TGC/CGC/AAT/CGA/G
KpnupC2	GAGTCA**GGATCC**ATG/GCG/GCC/TTA/TTG/ACT/TGC/GAA/AAG	GATTAT**GGGCC/C**AG/CAC/CAG/CCC/TGC/GAA/GCT/GG
KpvcCNT	GAGTCA**GGATCC**ATG/CAA/ATC/CTC/ATG/GGA/CTT/ATC/GGC/ATG	GATTAT**GGGCC/C**AG/GGC/GAG/AAA/AAC/TCC/GGC/GAT/G
KpnupG	GACGCT**GGATCC**ATG/AAT/CTC/AAG/CTG/CAG/CTC/AAA/ATA/CTG/TC	GATTAT**GGGCC/C**TC/GCT/CTG/CTG/TGC/TGC/GGT/CGG/TTG

Sequences are shown in the 5′⟶3′ order with restriction sites in bold and codons separated by forward slashes.
