# Supplementary figures and images for: Elucidation of the Gemcitabine Transporters of Escherichia coli K-12 and Gamma-Proteobacteria Linked to Gemcitabine-Related Chemoresistance
Source: Int J Mol Sci. 2024 Jun 27;25(13):7012. doi: 10.3390/ijms25137012 (PMC11241209; doi:10.3390/ijms25137012)

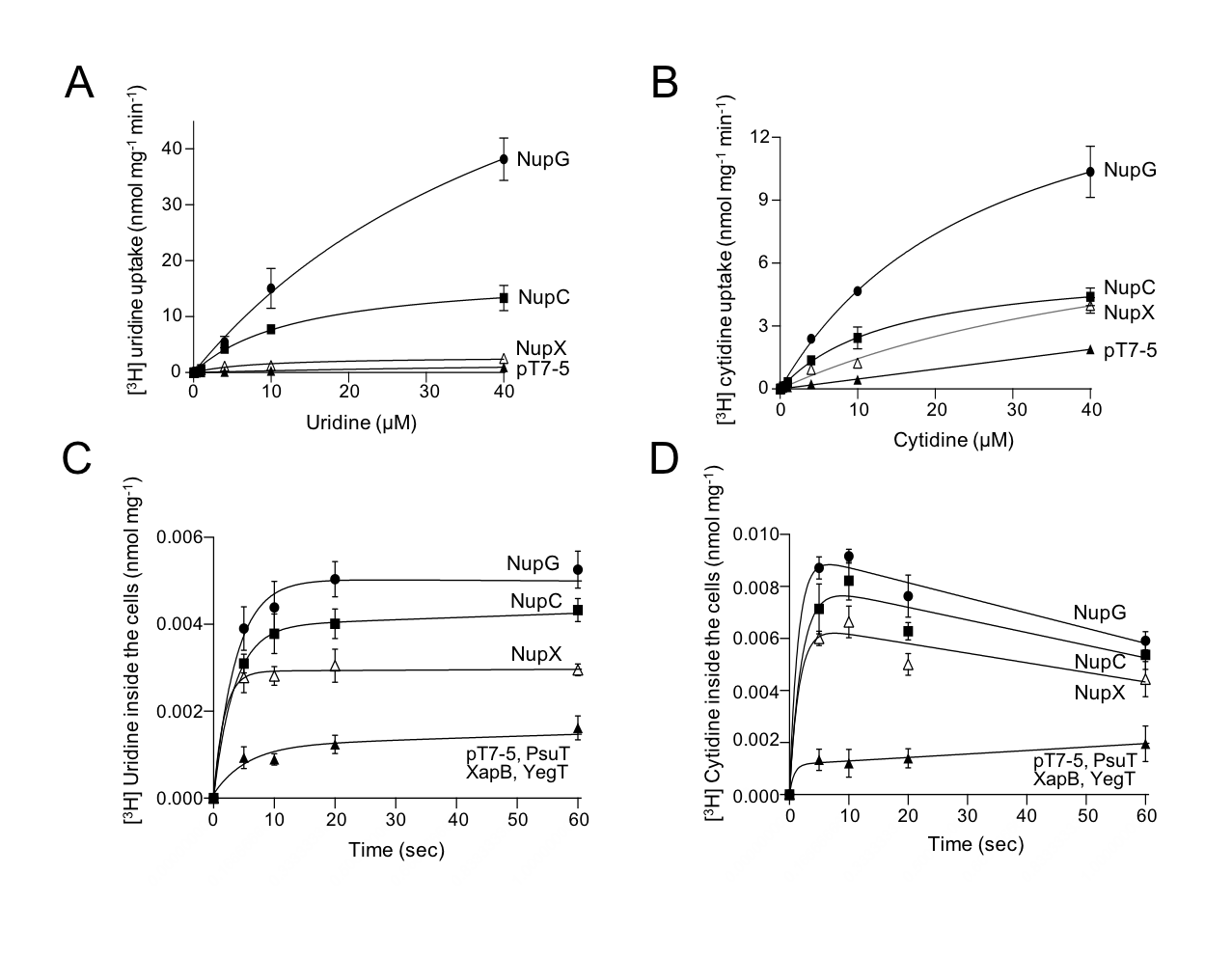

Supplement: Supplementary file 1 [file ijms-25-07012-s001.zip › Figure S1.png]

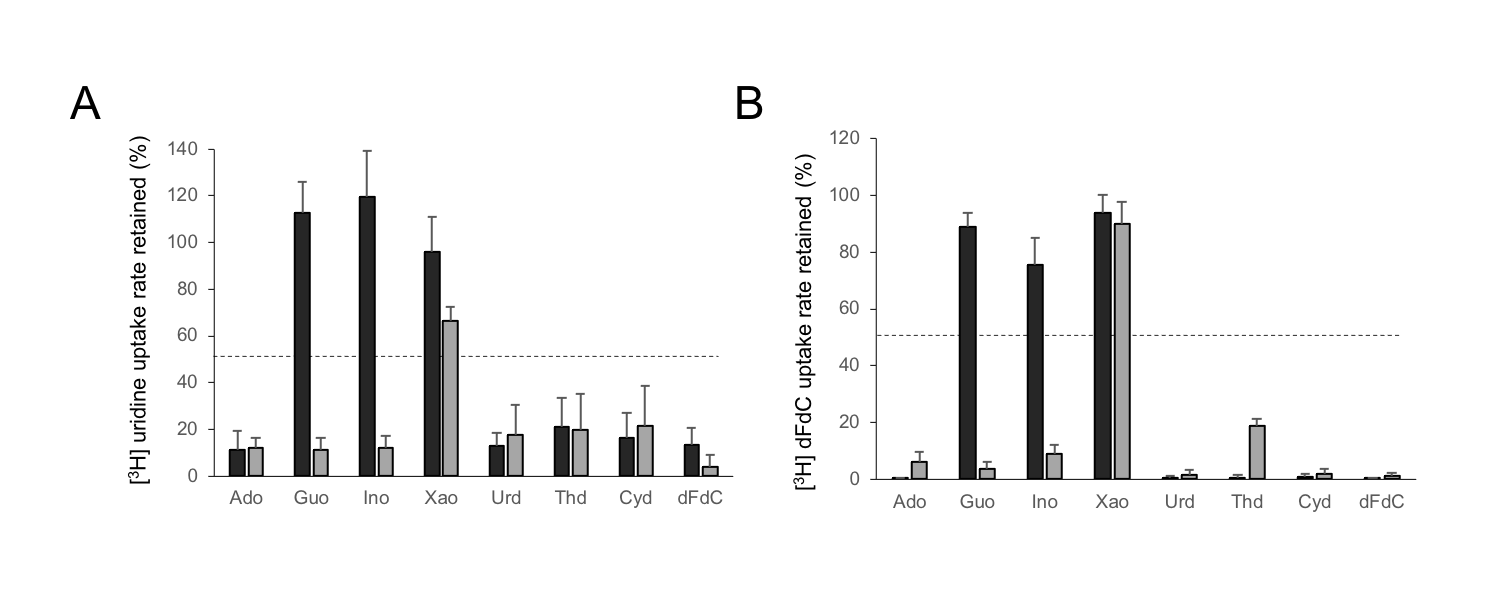

Supplement: Supplementary file 1 [file ijms-25-07012-s001.zip › Figure S2.png]

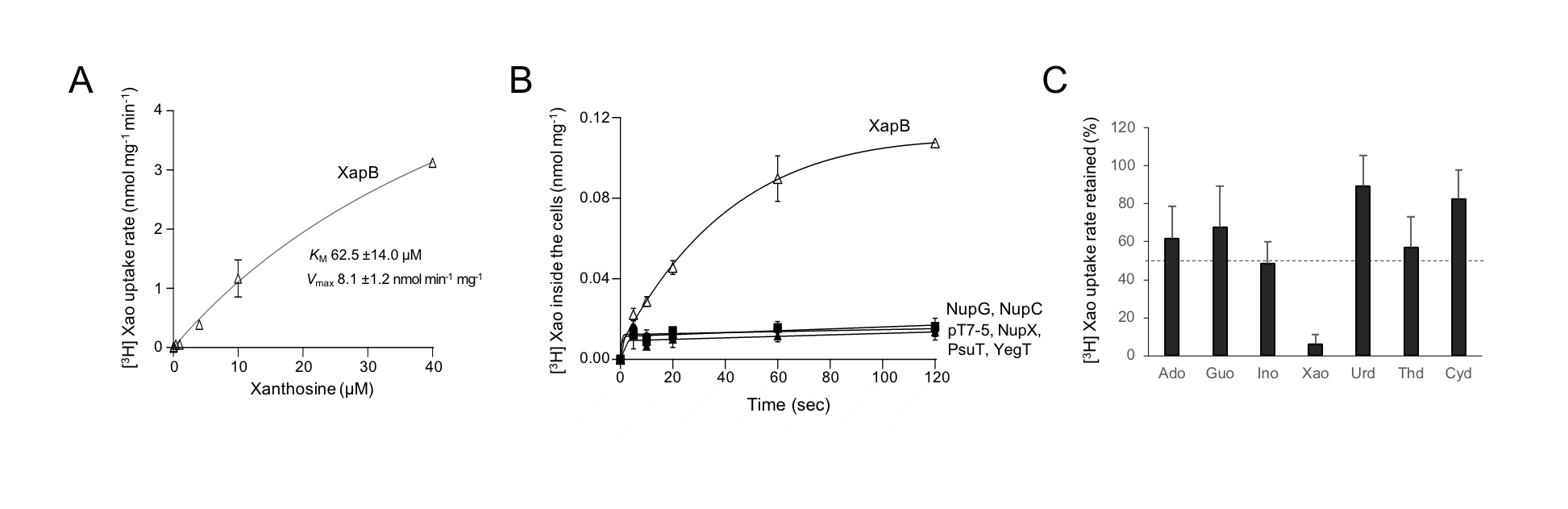

Supplement: Supplementary file 1 [file ijms-25-07012-s001.zip › Figure S3.png]

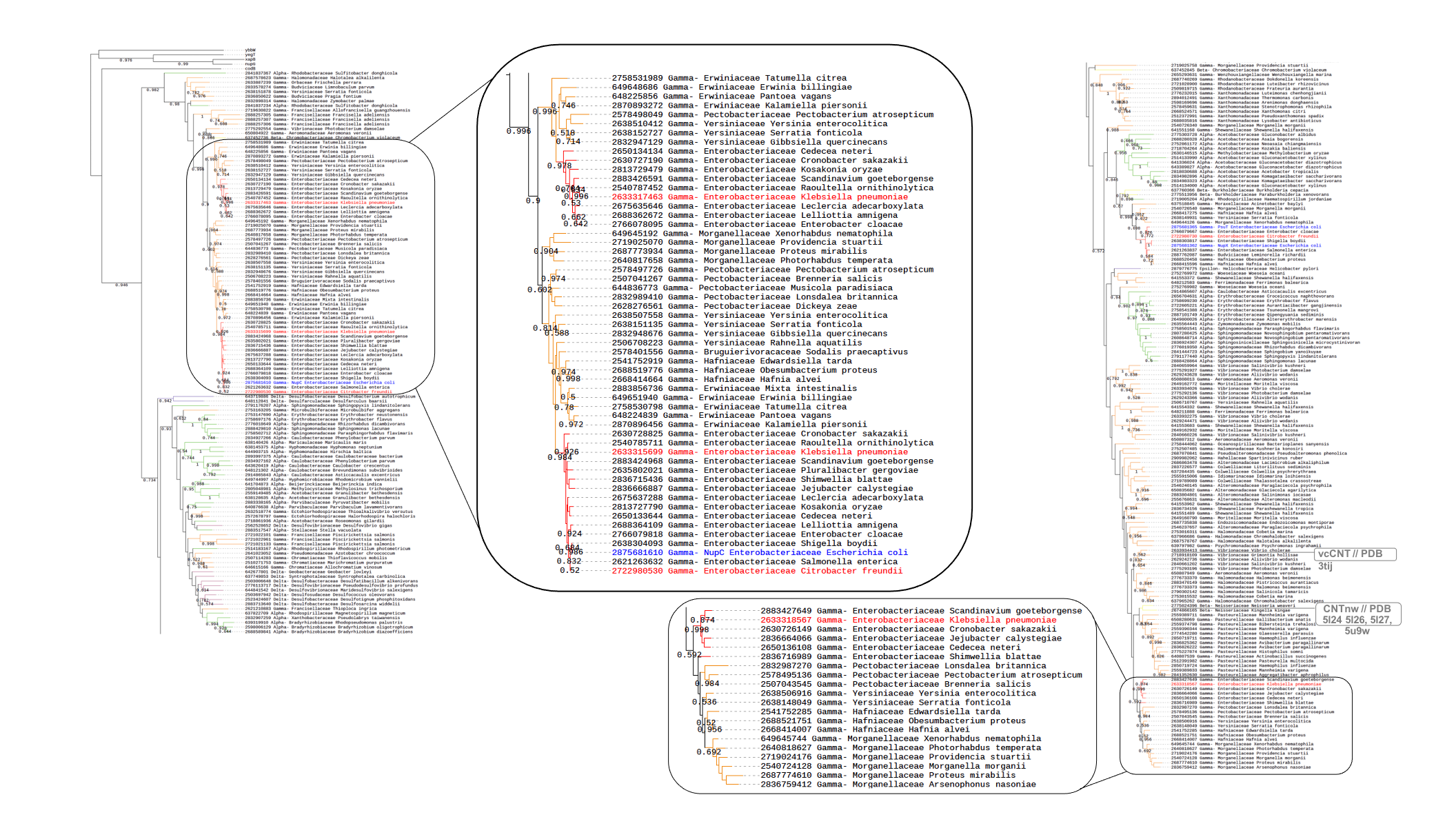

Supplement: Supplementary file 1 [file ijms-25-07012-s001.zip › Figure S4.png]

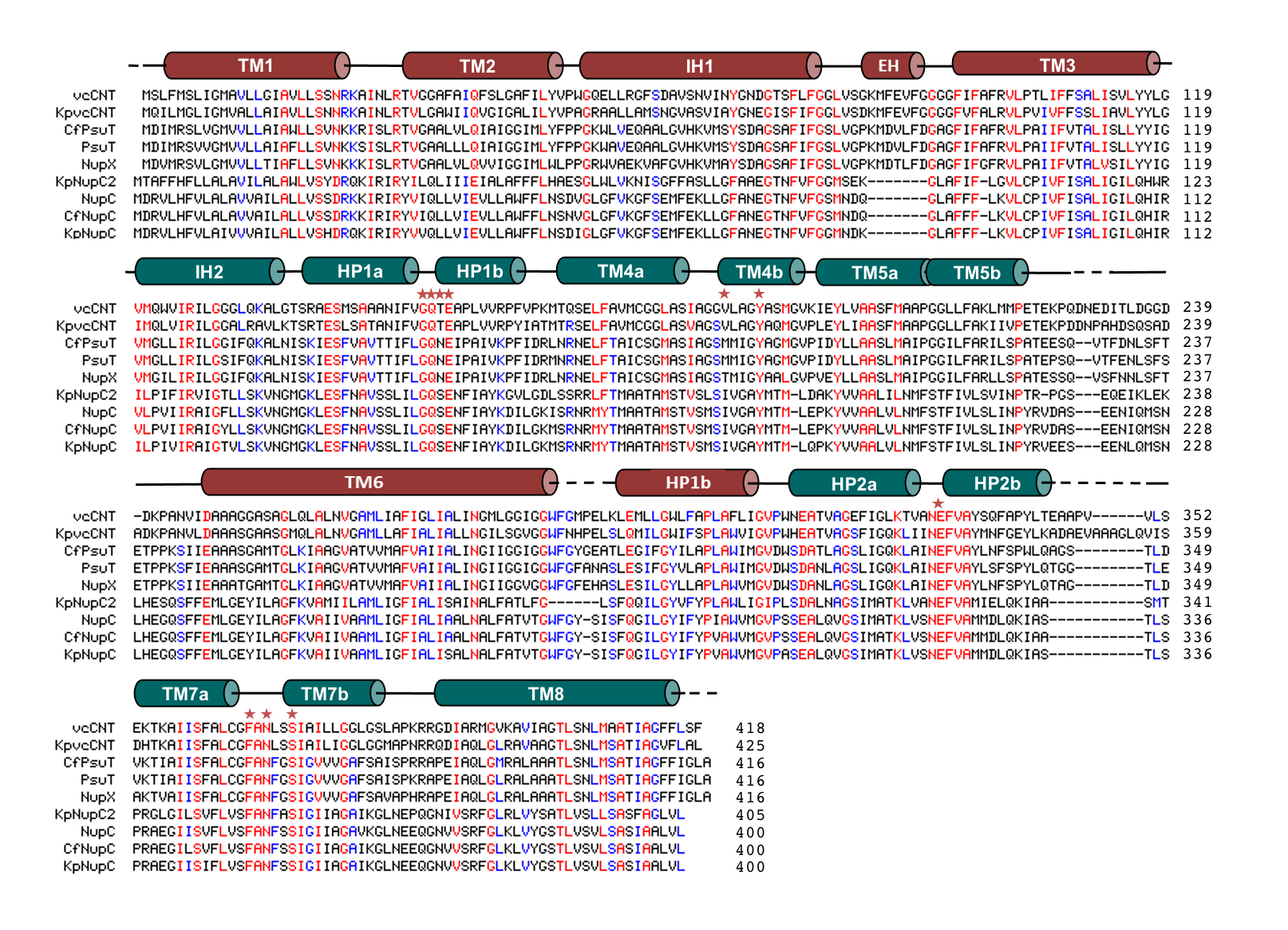

Supplement: Supplementary file 1 [file ijms-25-07012-s001.zip › Figure S5.png]

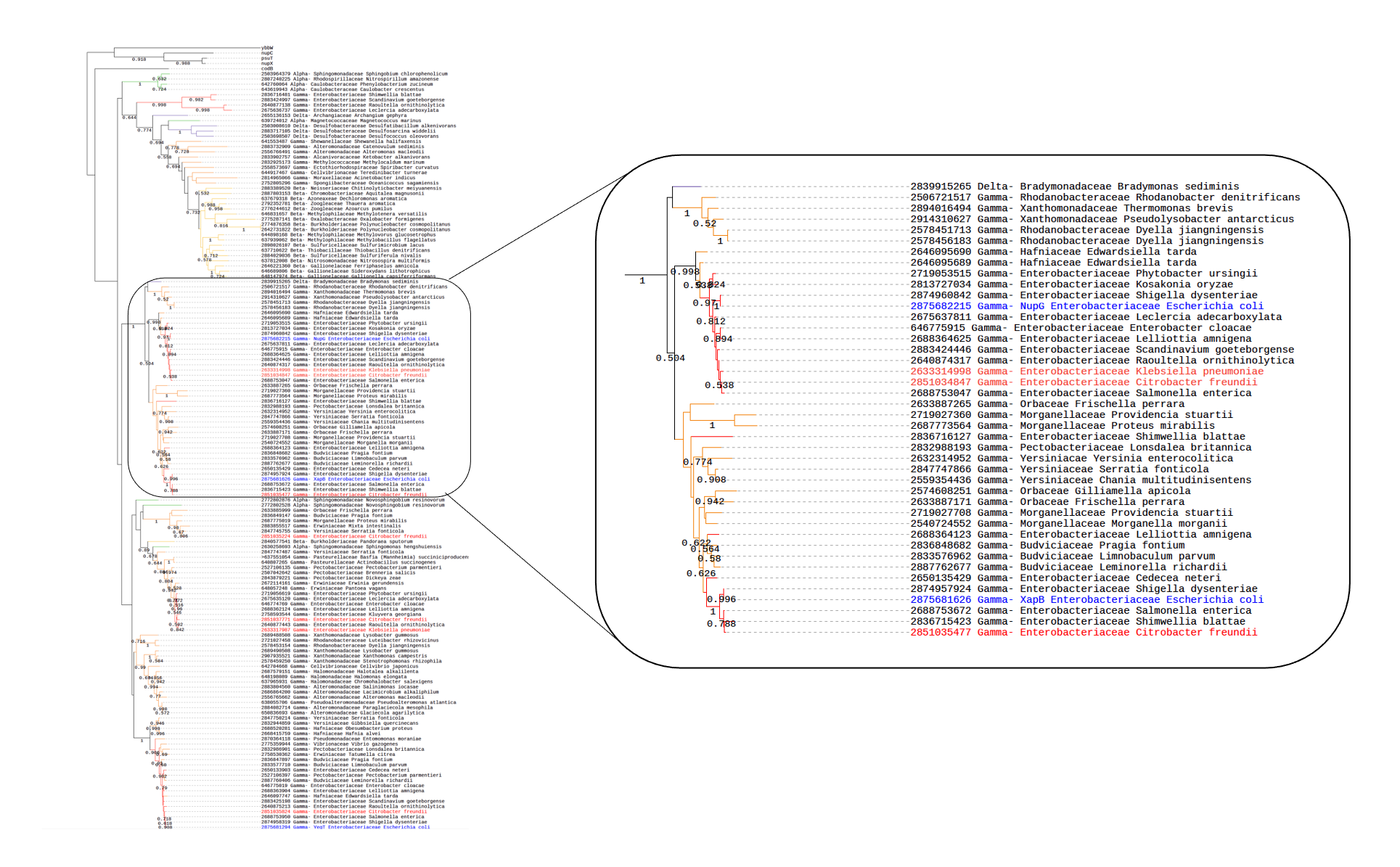

Supplement: Supplementary file 1 [file ijms-25-07012-s001.zip › Figure S6.png]

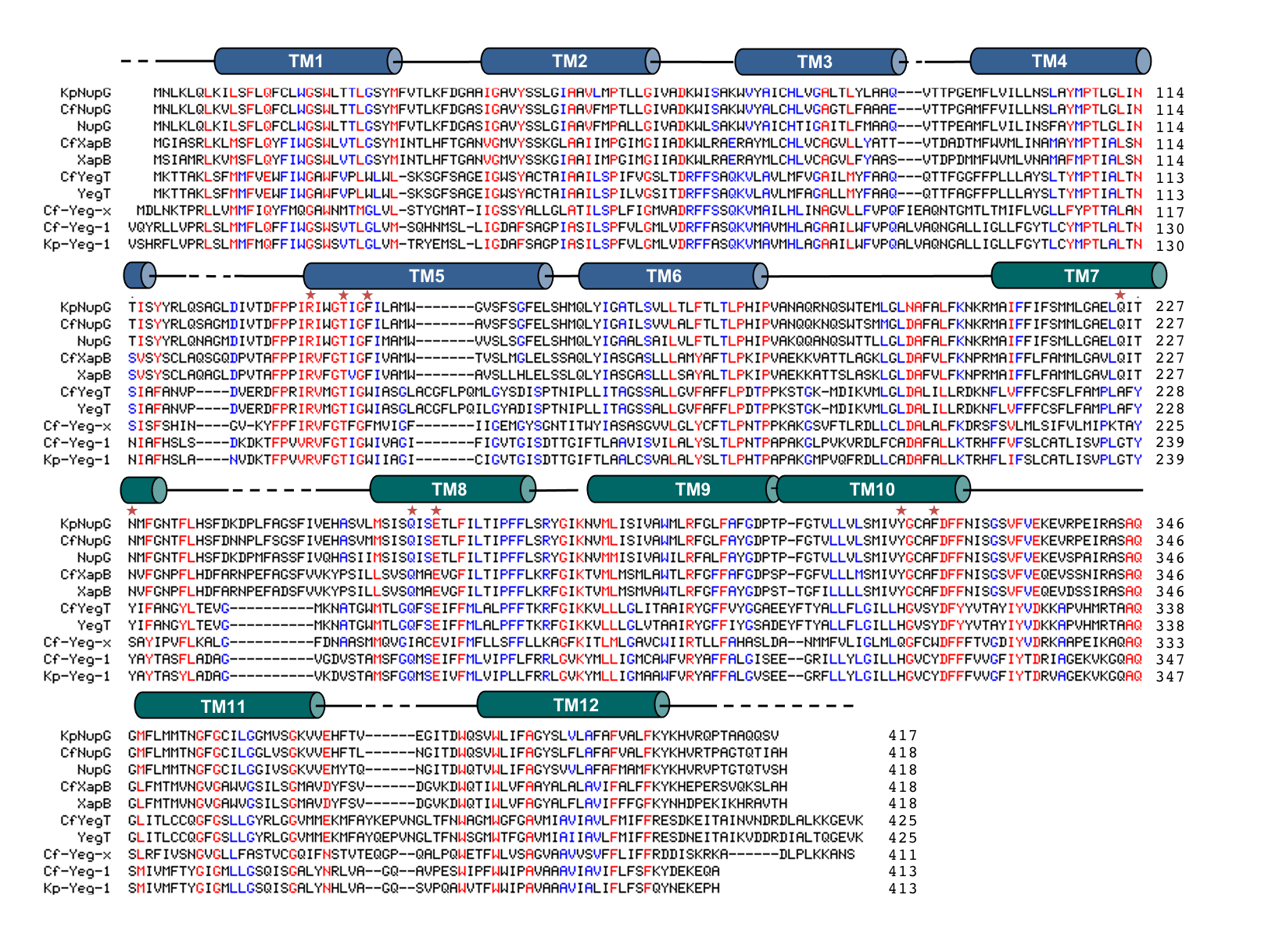

Supplement: Supplementary file 1 [file ijms-25-07012-s001.zip › Figure S7.png]

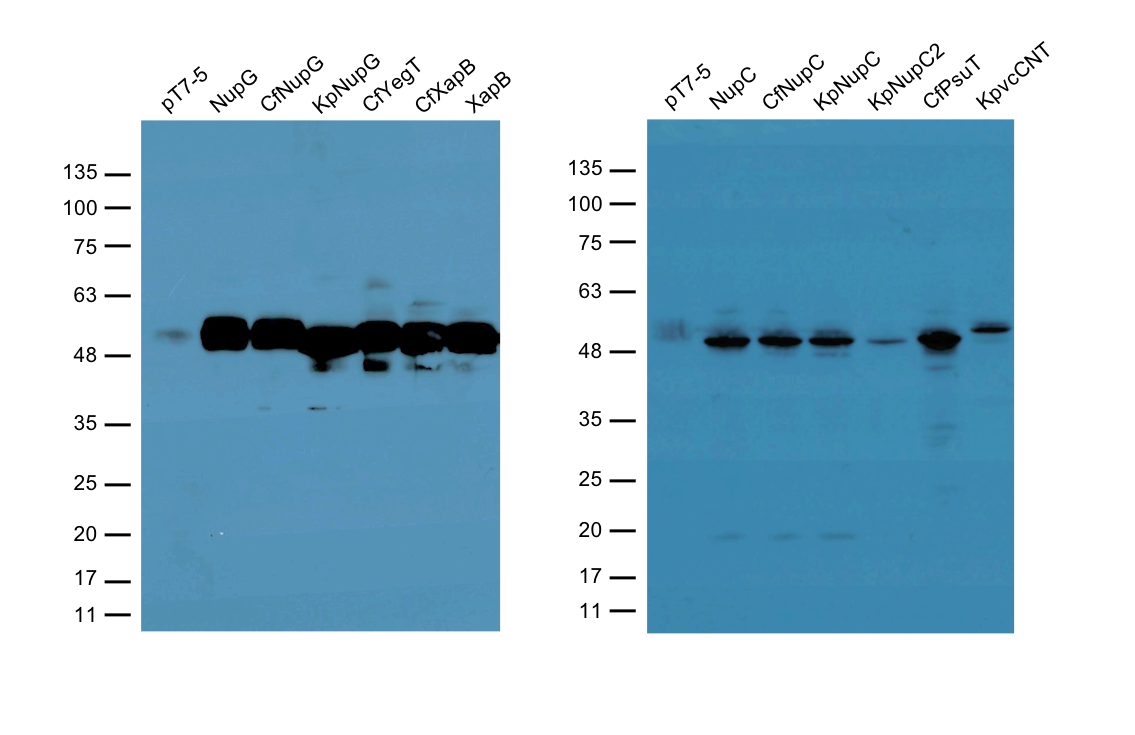

Supplement: Supplementary file 1 [file ijms-25-07012-s001.zip › Figure S8.png]

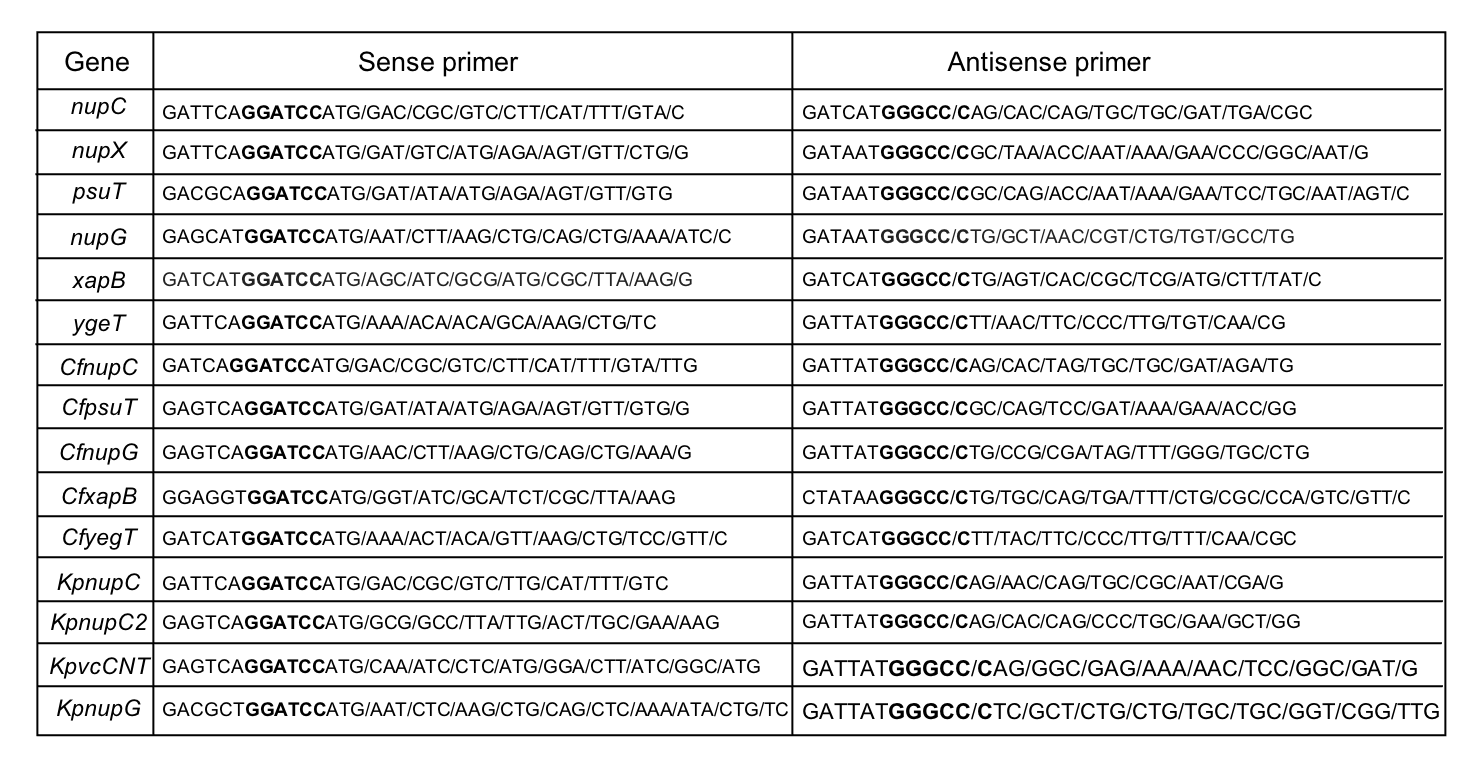

Supplement: Supplementary file 1 [file ijms-25-07012-s001.zip › Table S3.png]
